# Comparison of histological procedures and antigenicity of human post-mortem brains fixed with solutions used in gross anatomy laboratories

**DOI:** 10.3389/fnana.2024.1372953

**Published:** 2024-04-10

**Authors:** Eve-Marie Frigon, Amy Gérin-Lajoie, Mahsa Dadar, Denis Boire, Josefina Maranzano

**Affiliations:** ^1^Department of Anatomy, University of Quebec in Trois-Rivieres, Trois-Rivieres, QC, Canada; ^2^Department of Psychiatry, Douglas Research Center, McGill University, Montreal, QC, Canada; ^3^Department of Neurology and Neurosurgery, McConnell Brain Imaging Center, Montreal Neurological Institute, McGill University, Montreal, QC, Canada

**Keywords:** fixation, human brain, post-mortem, histology, antigenicity, anatomy laboratories, brain bank, immunohistochemistry

## Abstract

**Background:**

Brain banks provide small tissue samples to researchers, while gross anatomy laboratories could provide larger samples, including complete brains to neuroscientists. However, they are preserved with solutions appropriate for gross-dissection, different from the classic neutral-buffered formalin (NBF) used in brain banks. Our previous work in mice showed that two gross-anatomy laboratory solutions, a saturated-salt-solution (SSS) and an alcohol-formaldehyde-solution (AFS), preserve antigenicity of the main cellular markers (neurons, astrocytes, microglia, and myelin). Our goal is now to compare the quality of histology and antigenicity preservation of human brains fixed with NBF by immersion (practice of brain banks) vs. those fixed with a SSS and an AFS by whole body perfusion, practice of gross-anatomy laboratories.

**Methods:**

We used a convenience sample of 42 brains (31 males, 11 females; 25–90 years old) fixed with NBF (N = 12), SSS (N = 13), and AFS (N = 17). One cm^3^ tissue blocks were cut, cryoprotected, frozen and sliced into 40 μm sections. The four cell populations were labeled using immunohistochemistry (Neurons = neuronal-nuclei = NeuN, astrocytes = glial-fibrillary-acidic-protein = GFAP, microglia = ionized-calcium-binding-adaptor-molecule1 = Iba1 and oligodendrocytes = myelin-proteolipid-protein = PLP). We qualitatively assessed antigenicity and cell distribution, and compared the ease of manipulation of the sections, the microscopic tissue quality, and the quality of common histochemical stains (e.g., Cresyl violet, Luxol fast blue, etc.) across solutions.

**Results:**

Sections of SSS-fixed brains were more difficult to manipulate and showed poorer tissue quality than those from brains fixed with the other solutions. The four antigens were preserved, and cell labeling was more often homogeneous in AFS-fixed specimens. NeuN and GFAP were not always present in NBF and SSS samples. Some antigens were heterogeneously distributed in some specimens, independently of the fixative, but an antigen retrieval protocol successfully recovered them. Finally, the histochemical stains were of sufficient quality regardless of the fixative, although neurons were more often paler in SSS-fixed specimens.

**Conclusion:**

Antigenicity was preserved in human brains fixed with solutions used in human gross-anatomy (albeit the poorer quality of SSS-fixed specimens). For some specific variables, histology quality was superior in AFS-fixed brains. Furthermore, we show the feasibility of frequently used histochemical stains. These results are promising for neuroscientists interested in using brain specimens from anatomy laboratories.

## 1 Introduction

Normal and pathological aging of the human brain are topics of extensive research in the neuroscientific community. However, *in vivo* research is limited since standard imaging techniques, such as magnetic resonance imaging (MRI), do not provide information on molecular and cellular changes in the brain (at the cellular level of microstructure). Therefore, histology remains the Gold Standard method to unveil cellular changes that directly reflect underlying physio-pathological mechanisms (Durand-Martel et al., 2010; Cardoso et al., 2014; den Bakker, 2017). Ideally, brain tissue biopsies and surgical resections could be used for histological analysis [such as seen in Kishore et al. (2023), for example] and correlated with *in vivo* MRI, but these samples are rarely available for research given the invasiveness and risks associated with brain biopsies. Hence, post-mortem (i.e., *ex vivo*) research is the preferred methodological approach, as it provides a larger amount of cerebral tissue available for histology and study of the molecular and cellular brain alterations.

Brain banks typically provide small blocks or single slices of post-mortem tissue to researchers. However, gross anatomy laboratories could potentially be an additional source of post-mortem brain tissue by providing more numerous and larger brain tissue samples, and even complete brains. Currently, these brains are not used in neuroscientific research since they are preserved with solutions optimized for the preservation of bodies dedicated to gross anatomy dissection, that differ in composition from the classic neutral-buffered formalin solution (NBF) used in brain banks (Vonsattel et al., 2008; Beach et al., 2015; Carlos et al., 2019). Formaldehyde, along with alcohol, are the two most widely used chemicals for their antiseptic and antibacterial properties, and their capacity to reduce autolysis and therefore prevent tissue decomposition (Fox et al., 1985; Brenner, 2014; Musiał et al., 2016; Shetty et al., 2020; Martins-Costa et al., 2022). They also increase cross-linking of the proteins, affecting the antigens’ conformation (Puchtler and Meloan, 1985; Beach et al., 1987; Dawe et al., 2009; van Essen et al., 2010; Birkl et al., 2016). Additionally, formaldehyde hardens and distorts the tissue (Mouritzen Dam, 1979; Weisbecker, 2011). Furthermore, formaldehyde has been proven carcinogenic (Fisher, 1905; Frølich et al., 1984; Raja and Sultana, 2012; Thavarajah et al., 2012; Musiał et al., 2016) and consequently, this has led to the search for alternative solutions in anatomy laboratories to reduce formaldehyde’s harmful effects.

For instance, anatomy departments, such as ours at the University of Quebec in Trois-Rivières, have developed and use other fixative solutions that combine various chemicals, such as a saturated salt solution (SSS) (Coleman and Kogan, 1998; Hayashi et al., 2016), and an alcohol-formaldehyde solution (AFS), which contain ethanol, phenol, glycerol, and isopropylic alcohol, and a minimal concentration of formaldehyde. Comparative studies across these new fixative solutions have been exclusively anatomical (i.e., focused on the tissue flexibility and color, ease of dissection, lifespan of the specimens after dissection (i.e., time before contamination or decomposition), health hazards, costs, etc.) (Coleman and Kogan, 1998; Hayashi et al., 2014; Gulati et al., 2020; Shetty et al., 2020; Martins-Costa et al., 2022). However, to our knowledge, there are no studies on the ability of these solutions to preserve brain tissue adequately for (immuno)-histochemistry procedures. Instead, previous studies have focused on MRI (Schramek et al., 2013; Benet et al., 2014; Kanawaku et al., 2014; Maranzano et al., 2020; Nazemorroaya et al., 2022), other organs (Hopwood et al., 1989; Hammer et al., 2012; Cabrera et al., 2017; Lombardero et al., 2017; Rahman et al., 2021), or solutions with different chemical compositions than the SSS and the AFS currently employed by our laboratory (Hammer et al., 2012; Tomalty et al., 2019; Rahman et al., 2021). Therefore, these studies were limited with regards to information on tissue quality and antigen preservation in brain specimens fixed with such solutions, which is crucial to decide whether these brains are suitable for neuroscientific research.

Our previous work in mice showed that two solutions used in human gross anatomy laboratories (i.e., SSS and AFS), and the standard solution used in brain banks (i.e., NBF), preserved antigenicity of markers of the four main brain cell populations (i.e., neurons, astrocytes, microglia, and myelin of oligodendrocytes). Our work also showed that histological sections of brains fixed with SSS were difficult to manipulate and of poor quality but were still serviceable for immunohistochemical (IHC) and immunofluorescence (IF) analyses (Frigon et al., 2022). These promising results in mice might have been attributed to the lack of confounding variables that inevitably affect human samples (i.e., post-mortem delay, lengthy fixation period, variable ages of the donors, potential presence of vascular disease). Besides, human and mice brain characteristics are not alike (Ventura-Antunes et al., 2013), and as such, fixation quality through vascular perfusion of the specimens might not be comparable. Furthermore, as cellular densities differ (Herculano-Houzel et al., 2011; Ventura-Antunes et al., 2013; Loomba et al., 2022), we cannot deduce whether antigenicity and histological quality are preserved in human brain tissue in a similar fashion. Also, conclusive evidence on whether common histochemical stains, typically used for diagnostic purposes and neuropathology research, are feasible on brains fixed with solutions such as SSS or AFS is lacking. Therefore, several histological procedures need to be explored in post-mortem human brain tissue fixed with solutions typically used by anatomists.

The goal of the present study is to compare the quality of histological outcomes applied to human brains fixed using brain banks’ techniques (i.e., fixation by immersion in NBF) to those fixed with SSS or AFS by perfusion as applied in gross anatomy laboratories. We compared the ease of manipulation of the tissue, the tissue quality, the IHC labeling quality of four antigens of interest (neurons (anti-NeuN), astrocytes (anti-GFAP), microglia (anti-Iba1) and oligodendrocytic myelin protein (anti-PLP)) and the quality of various histochemical stains (i.e., Cresyl Violet, Luxol Fast Blue, Prussian blue and Bielchowsky’s) across the three experimental groups.

## 2 Methods

### 2.1 Population

We used a convenience sample of 42 human brains (NBF: *N* = 12; SSS: *N* = 13; AFS: *N* = 17) from our body donation program (Université du Québec à Trois-Rivières, Canada) as well as the Douglas-Bell Canada Brain Bank (Douglas Mental Health University Institute, McGill University, Montréal, Canada). Prior to their death, the donors to the anatomy laboratory provided consent on body donation and sharing of medical information for teaching and/or research purposes. The study was approved by the University’s Ethics Subcommittee on Anatomical Teaching and Research. Donors from the Brain Bank were obtained in collaboration with the Quebec Coroner’s Office and with informed consent from the nearest relative, following the guidelines of the Douglas Research Center Ethic Committee.

The median age at time of death of the donors was 76 years old (range: 25 to 90). The male to female ratio was 3:1. The case number, age, sex, cause of death, post-mortem delay to the fixation treatment (i.e., post-mortem interval) and delay until the histology processing of the tissue, are reported in Table 1.

**Table 1:** Specimen data.

Fixative	Sex	ID	Age	Cause of death	PMI (hours)	Histology processing delay (years)	Histology block of neocortex
NBF	F	1	47	Intoxication	39	4	Frontal
		2	62	Acute coronary syndrome	48.25	3	Frontal
		3	47	Suicide	43	2	Frontal
		4	28	Undetermined	39.5	2	Frontal
		5	32	Cardiac arrest by intoxication	73.25	1.5	Frontal
	M	6	53	Alzheimer disease	24	6	Frontal
		7	61	Metastatic lung cancer	24	9	Frontal
		8	85	Pulmonary edema	12	5	Frontal
		9	74	Lung cancer	3.25	1	Temporal
		10	52	Coronary thrombosis	38.5	5	Frontal
		11	42	Suicide	47	4	Frontal
		12	63	Cardiac arrest	47.75	1.5	Frontal
SSS	F	13	67	MPOC	36	2	Frontal
		14	75	Multiple sclerosis	27	2	Frontal
		15	84	Myocardial infarction	60	1	Parietal
		16	87	Pneumonia	9.5	1	Temporal
	M	17	44	Oligodendroglioma	24	2	Frontal
		18	80	Pneumonia	24	2	Frontal
		19	83	Heart failure	20	1	Temporal
		20	76	Pneumonia	6	3	Parietal
		21	25	Glioblastoma	42	1	Frontal
		22	64	Larynx cancer	7.5	3	Temporal
		23	87	Lung cancer	7.5	3	Parietal
		24	59	Severe malnutrition	24	3	Parietal
		25	85	Lung cancer	16.5	1	Frontal
AFS	F	26	64	Pneumonia, dementia	15.5	2	Temporal
		27	59	Metastatic lung cancer	18	9	Frontal
	M	28	82	Metastatic colon cancer	22	1	Temporal
		29	61	Metastatic lung cancer	24	9	Temporal
		30	90	Metastatic prostate cancer	37	2	Temporal
		31	88	Myocardial infarction	12	2	Frontal
		32	82	Pneumonia and infarction	48	10	Frontal
		33	86	Colon cancer	48	6	Frontal
		34	79	Tongue, throat, lung cancer	22	1	Frontal
		35	80	Alzheimer disease, pyelonephritis	12	4	Frontal
		36	82	Large-cell lymphoma	21	8	Frontal
		37	70	Lung cancer	10	15	Frontal
		38	86	Myocardial infarction	42	12	Parietal
		39	81	Lung cancer	21	8	Occipital
		40	88	Sepsis	31.5	13	Occipital
		41	84	Respiratory failure	22	5	Parietal
		42	78	Lung cancer	20	2	Frontal

PMI, Post-mortem interval; NBF, Neutral-buffered formalin solution; SSS, Saturated salt solution; AFS, Alcohol-formaldehyde solution; F, Female; M, Male.

### 2.2 Fixation procedure

All specimens were fixed with one of three solutions (chemical components are shown in Table 2). NBF-fixed brains were extracted fresh and then immersed in 4 liters of NBF following the brain bank technique and kept in formaldehyde until use. For SSS and AFS, the bodies were perfused with 25 L of one of the solutions through the common carotid artery using a pump (41 kPa) (Duotronic III, Hygeco International Products) which is the common embalming technique performed in gross anatomy laboratories (Maranzano et al., 2020). All the specimens were kept refrigerated until use. The brains were extracted, and 1 cm^3^ blocks (neocortex for gray matter and subcortical white matter) were taken, choosing an area that was macroscopically of good quality (no superficial tears, good color and texture) and not damaged during the extraction and arachnoid removal procedure.

**Table 2:** Fixative’s components.

NBF	SSS (Coleman and Kogan, 1998)	AFS
10% formalin	0.296% formaldehyde	1.42% formaldehyde
0.1 M PBS	36% NaCl	69% ethanol
	0.72% phenol	1.88% phenol
	2% glycerol	20.7% glycerol
	16% isopropylic alcohol	0.01% sodium acetate
		0.13% dettol

NBF, Neutral-buffered formalin solution; SSS, Saturated salt solution; AFS, Alcohol-formaldehyde solution; PBS, Phosphate-buffer saline.

### 2.3 Histology processing

Blocks were rinsed in 0.1 M PBS and immersed in 30% sucrose for 2 to 5 days (until the floating block sinks, which is the sign of appropriate sucrose diffusion into the tissue) for cryoprotection. Blocks were then frozen on dry ice before cutting 40 μm sections on a cryostat (Leica CM1950) at -19°C, that were either 1-mounted on 2% gelatin-subbed slides for the histochemical stains (Cresyl violet, Luxol Fast blue, Prussian blue and Bielchowsky’s), or 2-placed in well plate with PBS for subsequent free-floating IHC procedures. After IHC or histochemical staining (see the sections below), the sections were dehydrated in 5-min baths of increasing grades of ethanol (70, 95, 100%) and coverslipped using Eukitt.

### 2.4 Immunohistochemistry

Free-floating sections were rinsed three times for 5 minutes in 0.1 M PBS. They were then incubated in a 20% aqueous methanol solution with 0.5% H_2_O_2_ for 5 minutes to quench endogenous peroxidase. After another round of rinses (3x5minutes in 0.1 M PBS), sections were incubated in blocking solution for 2 hours (3% Normal Donkey Serum; 0.5% Bovine Serum Albumin; 0.3% Triton X-100 in 0.1 M PBS), followed by the primary antibodies’ (Table 3) incubation at 4°C in the same blocking solution overnight. Sections were rinsed again (3x5minutes in 0.1 M PBS) before a two-hour incubation in donkey anti-rabbit biotinylated secondary antibody (1:500) at room temperature in the same blocking solution. After another rinse, sections were incubated in an avidin-biotin complex (ABC) kit for 30 minutes in the dark (VECTASTAIN^®^ Elite^®^ ABC-HRP Kit, Vector Laboratories, catalog#VECTPK6100). Sections were then rinsed for 5 minutes in TRIS-buffered saline (TBS) (0.05 M, 0.9% NaCl) before the final incubation in 0.07% diaminobenzidine (DAB) (Sigma, #D5905-50TAB) with 0.024% H_2_O_2_ in TBS for 10 minutes.

**Table 3:** Immunohistochemical antibodies.

Primary antibodies	Animal	Laboratory	Dilution	Code
Anti-Neuronal nuclei (NeuN)	Rabbit	Abcam	1:3000	Ab177487
Anti-Glial fibrillary acidic protein (GFAP)	Rabbit		1:1000	Ab68428
Anti-Ionized calcium binding adaptor molecule1 (Iba1)	Rabbit		1:1000	Ab178847
Anti-Myelin proteolipid protein (PLP)	Rabbit		1:3000	Ab254363
Secondary antibody	Animal	Laboratory	Dilution	Code
Anti-Rabbit Biotinylated	Donkey	NovusBio	1:500	NBP1-75274

### 2.5 Histochemical stains

All specimens were also processed for four histological stains, commonly used in brain histopathology, namely 1-Cresyl Violet (endoplasmic reticulum of the cell nuclei, Nissl bodies), 2-Luxol Fast Blue (myelin), 3-Prussian blue (iron stain with a neutral red counterstain for Nissl bodies), and 4-Bielchowsky’s (neurofibrils and axons). Prior to the staining, sections were defatted in Xylene for 30 min and hydrated in increasing alcohols −100% Ethanol 2 × 1 min; 3–95% Ethanol 2 × 1 min; 70% Ethanol 2 × 1 min; H_2_O_d_ 1 min. After the staining (see protocols below), a dehydration process was applied using the same baths, but in a reverse order.

#### 2.5.1 Cresyl violet

Cresyl violet (CV) stains the endoplasmic reticulum and the cellular nuclei in dark purple, which includes, in the brain, cell bodies of neurons and glial cells (Eilam-Altstädter et al., 2021). The protocol followed guidelines of US Army Inst Pathol (Luna and Armed Forces Institute of, 1968), in which the sections were hydrated first, submerged for 1 h15 in 0.01% Cresyl violet (Sigma Aldrich catalog #C5042) in a 0.1 M acetate buffer (Anachemia, AC-8218) and 0.1 M acetic acid (BDH 10001CU), and then dehydrated.

#### 2.5.2 Luxol fast blue

Luxol flast blue (LFB) stains myelin, and should be dark blue in the white matter, and lighter blue (or even clear) in the gray matter. We followed principal guidelines and adapted the protocol from Pathology Center-Histological methods for CNS^1^ (Tokyo Metropolitan Institute of Medical Science) for this staining. The sections were incubated 2 h at 45°C in 0.1% Luxol Fast Blue (Solvent blue 38, Sigma Aldrich, catalog #S3352) in 95% Ethanol, then rinsed in H_2_O_d_. The differentiation (clearing over-dye) was performed in 0.05% Lithium carbonate (Sigma Aldrich, catalog #62470) for 1 min before immersion in 70% Ethanol for 10 min.

#### 2.5.3 Prussian blue

Prussian blue was performed to stain ferric iron in blue in combination with a Nuclear fast red counterstain which stains cell nuclei in pink. Iron Stain Kit (Abcam, ab150674) was used according to the manufacturer’s protocol.^2^ In order to assess the feasibility of this staining, we included cases diagnosed with Alzheimer’s disease in which we expected to detect iron (LeVine, 1997). Once validated, we proceeded to stain all the other specimens.

#### 2.5.4 Bielchowsky silver stain

Bielchowsky’s method is a silver staining protocol to label nerve fibers and neurofibrils in dark brown/black, and is commonly used to demonstrate neurofibrillary tangles and senile plaques in the central nervous system (Stahnisch, 2015). Our protocol was inspired from Bielchowsky’s Silver Stain protocol by Paul Polak and Douglas Feinstein, University of Illinois, Chicago (abcam) and IHC world^3^ and adapted as follows: sections were placed into a 10% Silver nitrate (Sigma Aldrich, catalog #209139) solution for 20 min at 40°C, following 3 rinses in water and another 20 min incubation at 40°C in the same silver nitrate solution that was titrated with high-concentrated ammonium hydroxide (ThermoScientific, catalog #423305000). Sections were then placed 1 min in a working developer solution (8 drops of Stock Solution +8 drops of ammonium hydroxide +50 mL of water; Stock solution: 20 mL of 37% formaldehyde +0.5 g of Citric acid +2 drops of Nitric acid +100 mL of water), followed by a 1% ammonium hydroxide incubation. Sections were rinsed in water three times before a 5 min bath of 5% Sodium Thiosulfate (Sigma Aldrich, catalog #S7026).

### 2.6 Variables of interest

**Figure 1:**
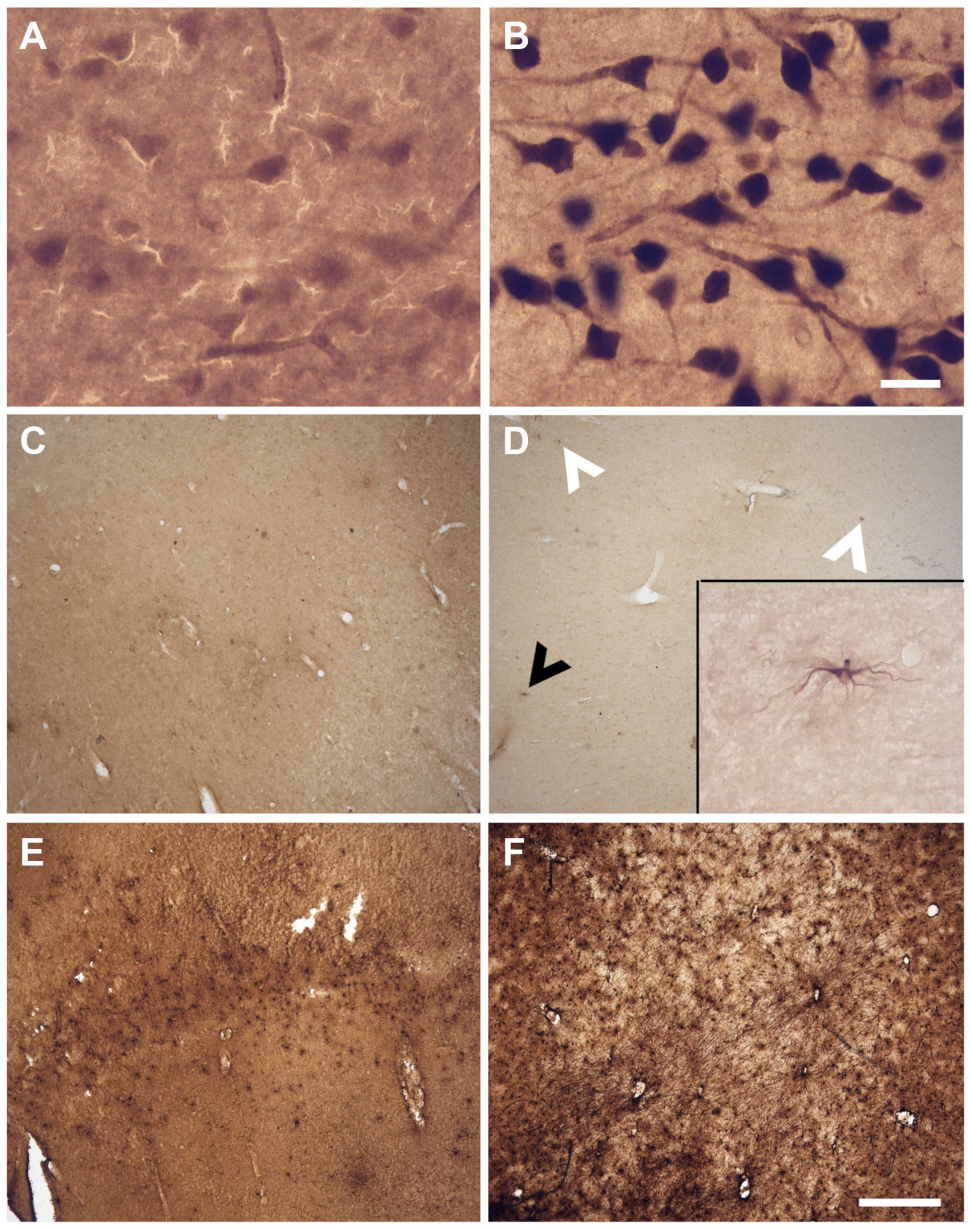
Photomicrographs of different criteria of the IHC categorical variables that were assessed. Photomicrographs (60X) of a fissured neuropil and shrunken and shriveled cells **(A)**, compared to a uniform neuropil with regular cell contour **(B)** (Section 2.6.2.1 and 2.6.2.2). Scale bar = 25 μm [valid for **(A,B)**]. Photomicrographs (4X) of the antigenicity preservation and distribution as an example using GFAP labeling, where we can see astrocytes that were completely absent **(C)**; isolated **(D)** [where arrowheads point out the cells, the black one being shown in the 60X insert; in patches **(E)**] or homogeneously distributed through the full analyzed sections **(F)** (Section 2.6.3). Scale bar = 500 μm [valid for **(C–F)**].

**Figure 2:**
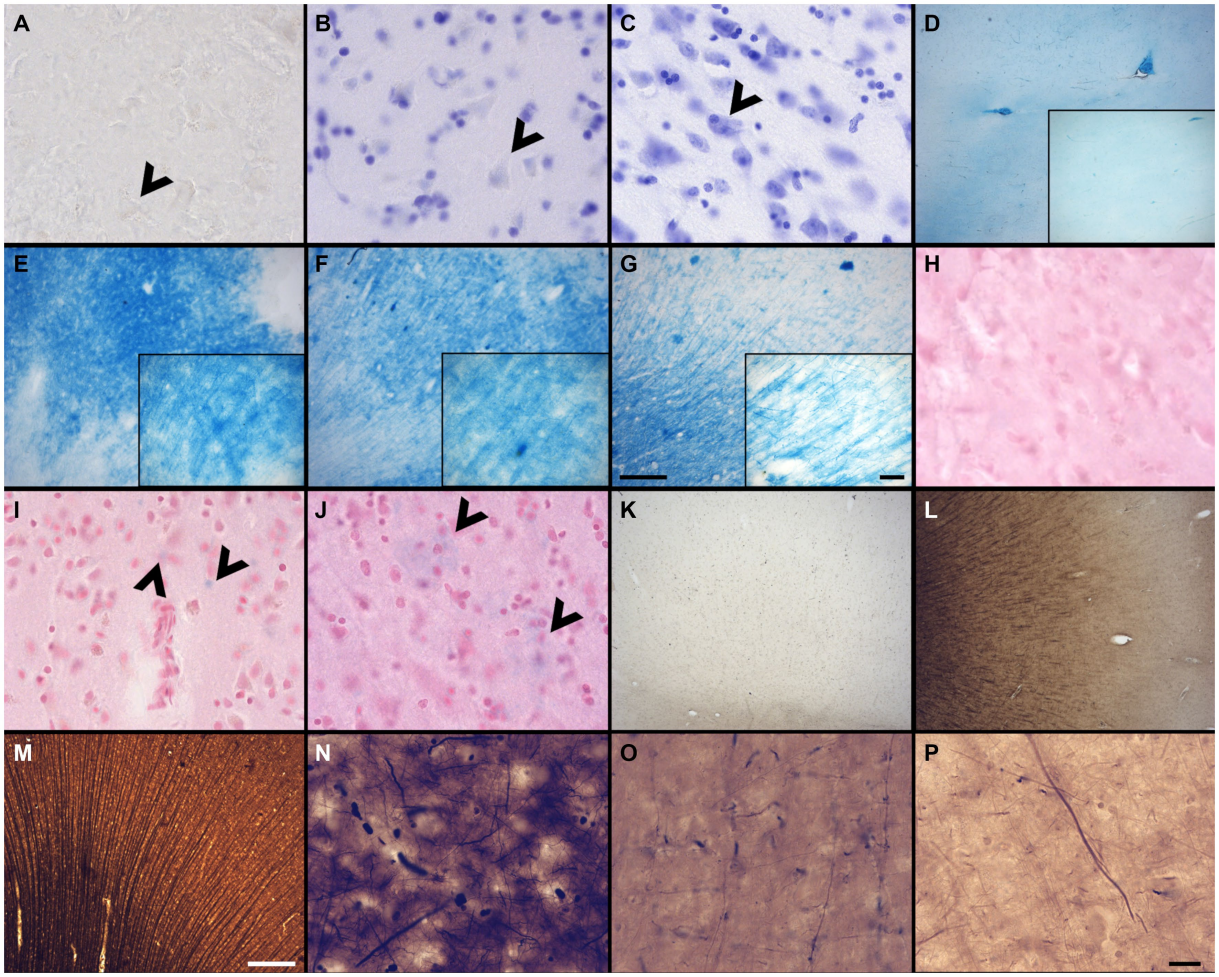
Photomicrographs of different criteria of the histochemical stains’ categorical variables that were assessed. Photomicrographs (60X) of an absent cresyl violet staining **(A)**, compared to when the neurons appear pale **(B)**, or strongly labeled (dark) neurons **(C)** (Section 2.6.4.1). Arrowheads point out neurons. Photomicrographs (4X) of the distribution of fibers and white matter-gray matter contrasts of **(D)** no fibers and an heterogeneous blue stains; **(E)** heterogeneous fibers and differentiation; **(F)** homogeneous fibers, but bad differentiation; or **(G)** homogeneous fibers and good differentiation. Scale bar = 500 μm [valid for **(D–G)**]. Inserts in **(D–G)** show photomicrographs (20X) of the myelin fibers in the respective categories (Section 2.6.4.2). Scale bar = 100 μm [valid for **(D–G)** inserts]. Photomicrographs (60X) of an absent neutral red (counterstain of the Prussian blue) staining **(H)**, compared to when the neurons appear pale **(I)**, or strongly labeled (dark) neurons **(J)** (Section 2.6.4.3). Arrowheads show blue spots, representing iron accumulation. Photomicrographs (4X) of the fibers preservation in the Bielchowsky’s stain, where we can see absence of fibers **(K)**, heterogeneous labeling of fiber **(L)** or homogeneous distribution of fibers **(M)**. Scale bar = 500 μm [valid for **(K–M)**]. Photomicrographs (60X) of the background in Bielchowsky’s stain (Section 2.6.4.4) that could be affected by silver deposits and therefore be dark **(N)**, intermediate **(O)**, or light **(P)**. Scale bar = 25 μm [valid for **(A–C,H–J,N–P)**].

The sections were assessed for multiple variables using a brightfield microscope (Olympus BX51W1) controlled by Neurolucida software (MBF Bioscience). We scored the IHC and histochemical stains variables to create qualitative categories that were compared for the three groups (fixatives) by looking through all the sections that were available for each specific staining. All variables were scored by a rater having 5-years experience in histology procedures and microscopy analysis (EMF). Figure 1 shows scoring of the criteria for different IHC variables (tissue quality and antigenicity preservation) that were assessed, while Figure 2 shows scoring of the criteria used for the four histochemical stains (see Supplementary Table S1 for all detailed scores of each specimen).

#### 2.6.1 Ease of manipulation

The first variable of interest was assessed during the processing of sections, i.e., cutting, well transfers, mounting on slides. We used four signs to assess the ease of manipulation: 1-slice consistency (soft or firm), 2- presence of tears (yes/no), 3- slices sticking to the brush that was used to manipulate/mount the slice (yes/no), and 4- presence of rolling slices (yes/no). Scores ranged from: Very poor (4 signs present = 0), Poor (3 signs present = 1), Good (1 or 2 signs present = 2) to Very good (No sign present = 3).

#### 2.6.2 Tissue quality

We used two criteria to assess this variable, namely the neuropil uniformity and the cellular shape, since it reflects the integrity of the tissue and the cellular morphology, susceptible to be differentially affected by the different chemicals (Spencer and Bancroft, 2008; Troiano et al., 2009) and the histology delay, which was in some specimens longer than 5 years. These criteria were assessed in the NeuN stained sections by scanning through the thickness of sections with a 60X objective (60X, UPlanSApo 60x/1.40 Oil∞/0.17/FN26.5 UIS2).

##### 2.6.2.1 Neuropil uniformity

The tissue was of lower quality when there was presence of a fissured neuropil. Scores indicated: Fissured = 0 (Figure 1A), Uniform = 1 (Figure 1B).

##### 2.6.2.2 Cellular shape

The neurons showed signs of altered shape (i.e., lower quality) if they presented an irregular and shriveled contour (irregular). Scores indicated: Irregular (shrunken/shriveled) = 0 (Figure 1A), Regular (smooth) = 1 (Figure 1B).

#### 2.6.3 Antigenicity preservation

We looked at sections targeted for all the antigens to verify the cell distribution across the section, using a 4X objective to have a large field of view (4X, UPlanSApo 4x/0.16∞/−/FN26.5). Antigenicity preservation was scored from worse (reflecting destruction/cross-linking of the antigen) to best (reflecting ideal preservation of the antigen) as follows: 1-absence of labeling (Figure 1C); 2-heterogeneous distribution of the cells [presence of isolated cells (Figure 1D), 3-cells’ patches (Figure 1E) or 4-a homogeneous distribution (Figure 1F)]. This was assessed for all the immunolabeled sections of the four antigens of interest. Scores indicated: Absence = 0 (Figure 1C), Isolated cells = 1 (Figure 1D), Cell patches = 2 (Figure 1E), Homogeneous distribution = 3 (Figure 1F).

Specimens that showed no antigenicity for one or more antigens or heterogeneous distribution (i.e., isolated cells or cell patches) were reprocessed following an antigen retrieval protocol. To this end, sections were subjected to 20-min boiling-citrate buffer (free-floating) and then immersed into a cold-water bath for 10 min. The IHC procedures were then performed following the same protocols (section 2.4), and the sections were analyzed anew.

#### 2.6.4 Histochemical stains’ quality

We assessed four histochemical stains to evaluate the preservation of different cell populations in a non-immunological way.

##### 2.6.4.1 Cresyl violet

A quality Nissl stain shows a clear contrast between well stained neuronal cell bodies with clear cytoplasmic granulations and the clear surrounding neuropil. Glial cells were strongly labeled in all cases, but neurons were heterogeneous in their staining. Therefore, a high contrast between the stained neuronal cell bodies and neuropil was considered as indicative of quality. This was assessed at low (4X) and high (60X) magnification of all the labeled sections available. Scores indicated: No staining = 0 (Figure 2A), Pale neurons = 1 (Figure 2B), Dark neurons = 2 (Figure 2C).

##### 2.6.4.2 Luxol fast blue

Luxol fast blue staining is known to dye myelinated axons and the oligodendrocytes’ nuclei that produce this myelin sheath in the white matter of the brain (Lindberg and Lamps, 2018). LFB is frequently used in the diagnosis of multiple sclerosis, Alzheimer’s disease, or white matter hyperintensities (Bronge et al., 2002; Laule et al., 2008; Gouw et al., 2011; Kuhlmann et al., 2017; Laule and Moore, 2018; Humphreys et al., 2019; Ouellette et al., 2020; Roseborough et al., 2020; Selvaraji et al., 2022). This staining was therefore important to assess in brains fixed with other solutions. The LFB stain was assessed by the contrast between the white matter and gray matter at low (4X) and high (60X) magnification. The white matter should show homogeneous distribution of fibers and be dark blue and should be clearly differentiated from the gray matter that should be free of staining or light blue. Scores indicated: No fibers and heterogeneous blue stain = 0 (Figure 2D); Heterogeneous fibers and differentiation = 1 (Figure 2E), Homogeneous fibers but bad differentiation = 2 (Figure 2F) and Homogeneous fibers and good differentiation = 3 (Figure 2G).

##### 2.6.4.3 Prussian blue

Prussian blue staining (Perl’s stain) is known to reveal ferric iron deposits in the neuropil of the brain (Churukian, 2008; Guindi, 2018; Xiao et al., 2023). This staining is of interest in neurodegenerative diseases, small vessel diseases, or white matter pathology detected by MRI, in which brains may be affected by such iron deposits (Connor and Benkovic, 1992; Kruer, 2013; Roseborough et al., 2020; Dusek et al., 2022). Since iron deposits did not occur in all specimens, this was not assessed in this staining; only the feasibility of the iron revelation in brains fixed with the three solutions was considered. However, we assessed the neutral red staining of the Prussian blue counterstain in a similar fashion as the cresyl violet. Scores indicated: No staining = 0 (Figure 2H), Pale neurons = 1 (Figure 2I), Dark neurons = 2 (Figure 2J).

##### 2.6.4.4 Bielchowsky’s

Bielchowsky’s silver stain is known as a silver impregnation method for nervous tissue (Ogawa, 1990) which is used to visualize axons, neurofibrils and neurofibrillary tangles or neuritic plaques (Wisniewski et al., 1989; Love and Nicoll, 1992; Allsop, 2000; Switzer, 2000; Uchihara, 2007; Kovacs and Budka, 2010; Elobeid et al., 2014; Fan et al., 2018). The quality was assessed using two criteria: the fibers preservation/distribution and the background intensity.

##### 2.6.4.5 Fibers’ preservation and distribution

The stained sections were scanned at low (4X) magnification to obtain an overall field of view. The staining was considered of good quality when well-labeled, dark silver impregnated fibers were homogeneously distributed across the sections. Scores indicated: Absence = 0 (Figure 2K), Heterogeneous = 1 (Figure 2L), Homogeneous = 2 (Figure 2M).

##### 2.6.4.6 Background

Silver stains are known to use toxic chemicals and are capricious, unpredictable and difficult to perform (Ogawa, 1990; Litchfield and Nagy, 2001), since silver binds and stains to chemicals, slides, mounting medium, dust, etc. Therefore, the background level of staining was assessed since non-specific silver deposits could vary according to the fixative solution and other variable conditions. Therefore, we observed the presence or absence of silver deposits in the background using the 60X objective, in which the presence of silver spots decreases the contrast of the well-stained fibers against the background, and therefore, a poorer quality of this staining. Scores indicated: Dark = 0 (Figure 2N), Intermediate = 1 (Figure 2O), Light = 2 (Figure 2P).

### 2.7 Confounding variables

All variables mentioned above were also assessed in relation to two confounding variables: the post-mortem interval and the histology delay, to determine whether they might have affected the variables of interest. Raw data is shown in Table 1. Also, see Supplementary Table S1 for all detailed scores of each specimen, and Supplementary Figures S1, S2 for scatter dot plots of all the variables of interest in relation to the confounding variables.

#### 2.7.1 Post-mortem interval

The post-mortem interval (PMI) is the time elapsed between death of the donor and the administration of the fixative to the brain. The PMI could have an impact on the tissue quality since the brain could start to decompose prior to fixation. The acceptable PMI is 0 to 48 h, although in cases where the bodies have been refrigerated the interval may be extended an additional 24 h. To assess this variable, we created two categories, for shorter or longer PMI: Short = 0–24 h; Long = >24 h. Note: the choice of 24 h to divide the full sample aimed to produce two groups of similar size. If the limit had been set before 24 h (i.e., 6 or 12 h), the size of the Short PMI group would have been too small to allow statistical analyses.

#### 2.7.2 Histology processing delay

The histology delay (HD) corresponds to the time elapsed between the moment the brain was fixed (by perfusion in the anatomy laboratory), or from the moment it was immersed in NBF (brain bank technique), to the moment the histology procedures began. Since we used a convenience sample, this variable was not controlled for all specimens. Some brain blocks were obtained after the brains were used for years for teaching Anatomy laboratory, UQTR (and therefore immersed in 10% ethanol); immediately after the brains were extracted (if fixed by perfusion, UQTR), or after years of immersion in NBF (Douglas-Bell Canada brain bank). These differences may affect the brains differently by causing different levels of cross-linking of the proteins (i.e., antigens). Therefore, we assessed this confounding variable creating two categories, for shorter or longer HD: Short = 0–2 years; Long = >2 years. Note: the choice of 2 years to divide the full sample aimed to produce two groups of similar size. If the limit had been set later (i.e., 4 or 6 years), the size of the Long HD group would have been too small to allow statistical analyses.

### 2.8 Statistical analyses

All categorical variables described above were compared across the three fixative groups using Chi-square tests. All statistical analyses were adjusted for multiple comparisons using Bonferroni correction and were performed using SPSS statistics software (28.0.0 version).

To assess for confounding variables, we also used Chi-square tests to relate the variables of interest (section 2.6) to each confounding variable (section 2.7), separately in each fixative group (NBF; SSS and AFS).

## 3 Results

### 3.1 Ease of manipulation

**Figure 3:**
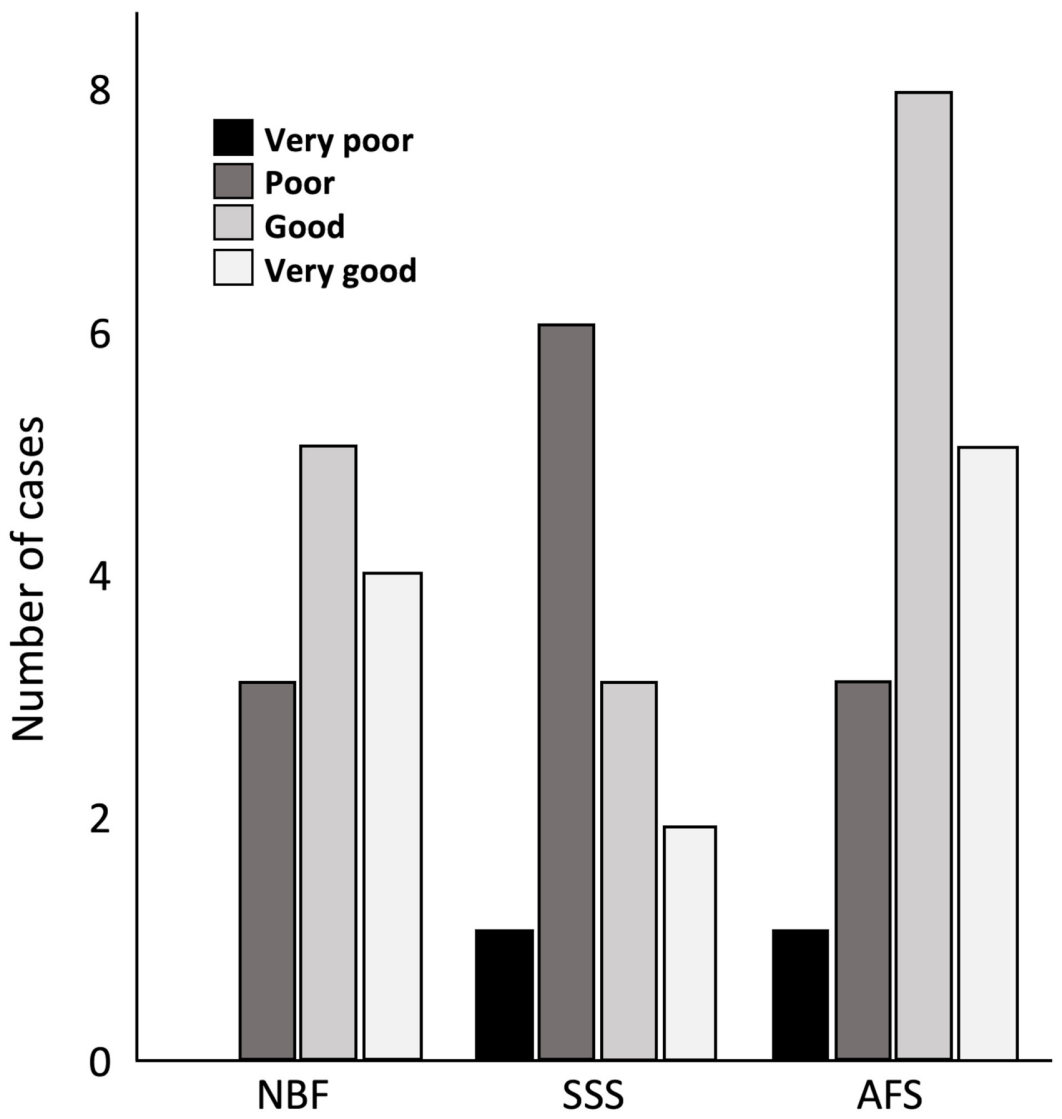
Bar charts of the ease of manipulation of the tissue. Very poor (black), 4 signs; Poor (dark gray), 3 signs; Good (light gray), 1 or 2 signs; Very good (white), no sign; NBF, neutral-buffered formalin; SSS, salt-saturated solution; AFS, alcohol-formaldehyde solution.

We found no statistically significant differences between treatment groups in the ease of manipulation scores, but we observed more cases fixed with SSS that scored poor for this criterion, while brains fixed with NBF never scored very poor for ease of manipulation. Also, a higher proportion of cases fixed with NBF and AFS scored good for this criterion (Figure 3).

### 3.2 Tissue quality

**Figure 4:**
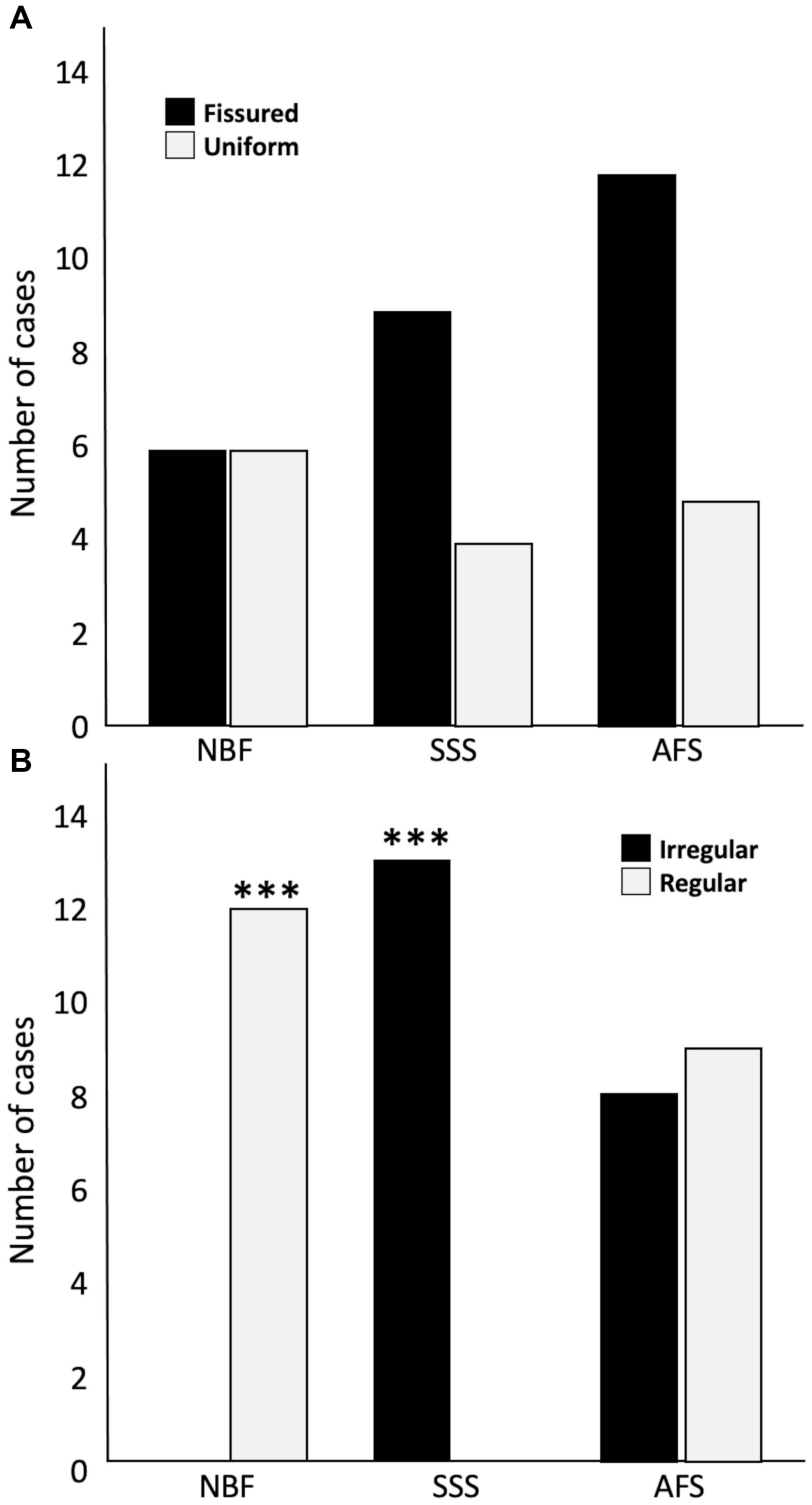
Bar charts of the tissue quality. Neuropil uniformity **(A)**, either fissured = 0 (black) or uniform = 1 (white), and cellular shape **(B)**, either irregular (shrunken and shriveled) = 0 (black) or regular (smooth) = 1 (white). ***, Significant after Bonferroni correction (*p* < 0.001); NBF, neutral-buffered formalin; SSS, salt-saturated solution; AFS, alcohol-formaldehyde solution.

Tissue quality was assessed according to two criteria, namely the uniformity of the neuropil and cellular shape. We found no significant difference in the frequency of occurrence of fissured neuropil across the three groups (Figure 4A). However, we found a significant difference in the frequency of scores for the cellular shape, between the three groups (*p* < 0.001). Regular cell contours were always observed in the NBF-fixed brains (*p* < 0.001), while irregular (shrunken and shriveled) cell contours were always found in SSS-fixed brains (*p* < 0.001) (Figure 4B).

### 3.3 Antigenicity preservation

**Figure 5:**
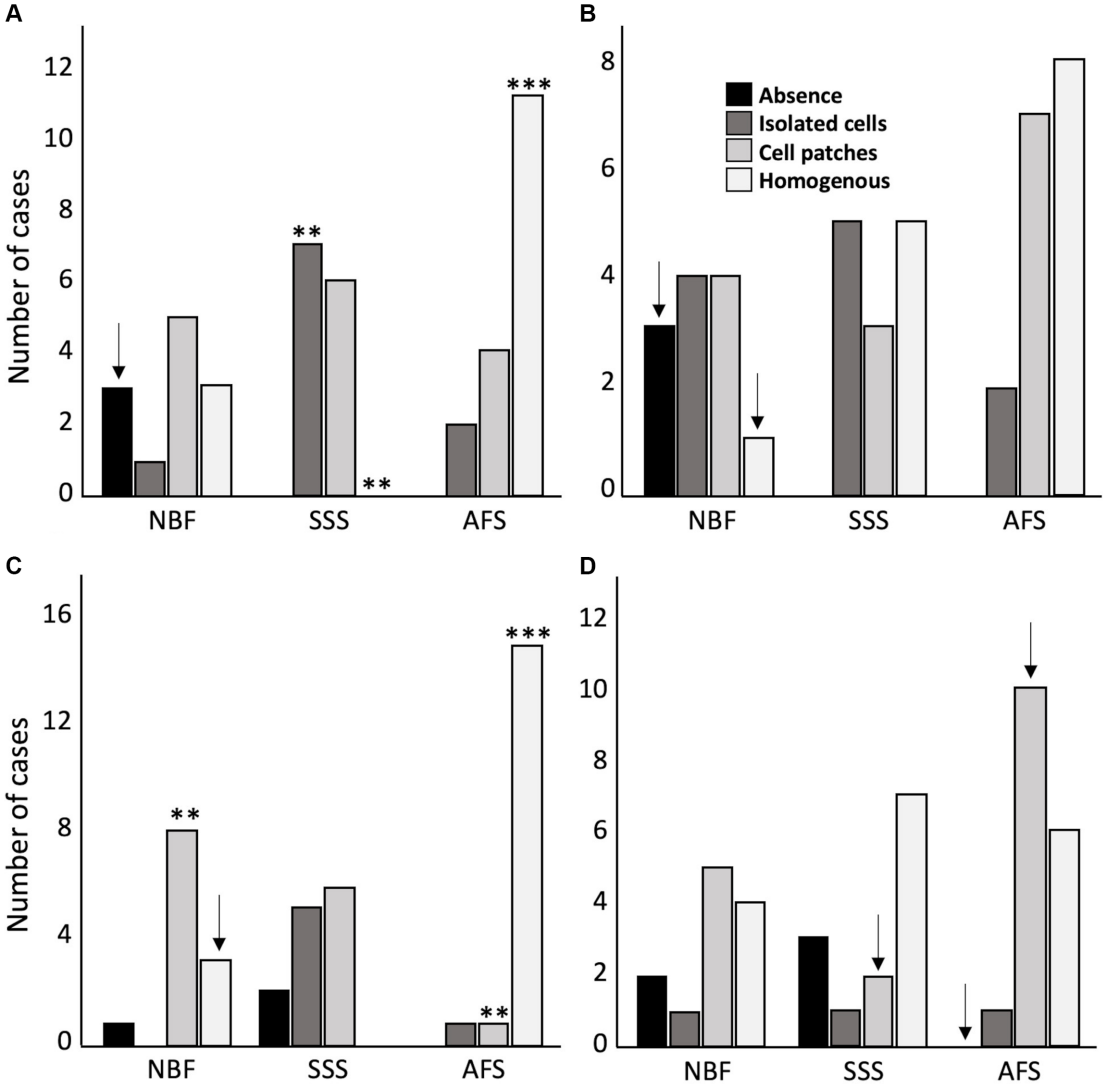
Antigenicity preservation and cellular distribution of the four main brain cell populations. Cellular distribution of Neuronal nuclei (NeuN) **(A)**, astrocytes (GFAP) **(B)**, microglia (Iba1) **(C)** and oligodendrocytic myelin fibers (PLP) **(D)**. No antigenicity = 0 (black); Isolated cells = 1 (dark gray); Cell patches = 2 (light gray); Homogeneous distribution = 3 (white). ** = Significant after Bonferroni correction (*p* < 0.01); *** = Significant after Bonferroni correction (p < 0.001); ↓ = Significant before Bonferroni correction; NBF = neutral-buffered formalin; SSS = salt-saturated solution; AFS = alcohol-formaldehyde solution.

The distribution of labeled cells of the four tested antigens was used to assess antigenicity preservation. A homogeneous distribution of NeuN labeling was significantly more frequent in AFS-fixed brains (*p* = 0.0003), whereas significantly higher occurrence of isolated labeled cells (*p* = 0.0019) and no homogeneous NeuN labeling (*p* = 0.0019) were observed in SSS fixed brains. NeuN antigenicity was preserved in most NBF-fixed specimens, except for three cases in which no labeled neurons were observed (*p* = 0.005; significant before a Bonferroni correction) (Figure 5A). Homogeneous distribution of GFAP labeling was observed in only one NBF-fixed specimen, and no labeled astrocytes were observed in three cases (*p* = 0.005; significant before a Bonferroni correction) (Figure 5B). Patchy Iba1 labeling occurred significantly more frequently (*p* = 0.0037) whereas homogeneous labeling of this marker occurred less frequently in NBF-fixed brains (*p* = 0.005; significant before a Bonferroni correction). Homogeneous distribution of Iba1 labeling was significantly more frequent, whereas patchy labeling was significantly less frequent in AFS-fixed brains (*p* < 0.001) (Figure 5C). Finally, PLP labeling was more frequently uniform (homogeneous or cell patches) in all AFS-fixed brains (more cases of cell patches: *p* = 0.046 before a Bonferroni correction), Conversely PLP labeling was absent in three SSS-fixed cases and two NBF-fixed cases (Figure 5D).

**Figure 6:**
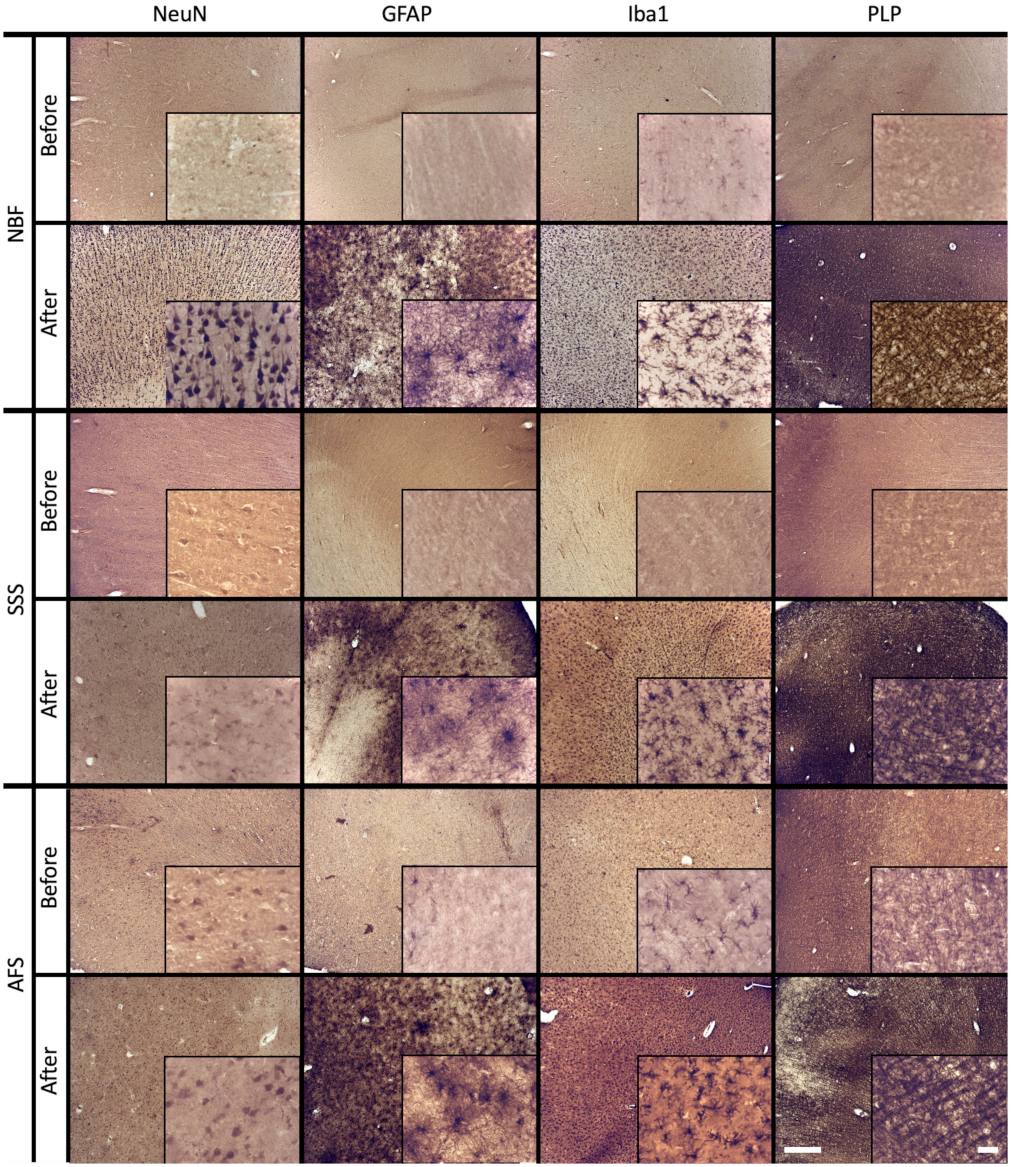
Before and after an antigen retrieval protocol in sections that showed poor antigenicity preservation. Photomicrographs (4X) (inserts are zoomed-in at 40X to see the cells) of cellular distribution of NBF-fixed cases (top), SSS-fixed cases (middle) and AFS-fixed cases (bottom line) that showed no antigenicity (before lines), while a homogeneous distribution is shown after an antigen retrieval protocol (after lines). Left scale bar is valid in all 4X photomicrographs = 500 μm. Right scale bar is valid in all inserts (40X) = 50 μm. NBF, neutral-buffered formalin; SSS, salt-saturated solution; AFS, alcohol-formaldehyde solution; NeuN, neuronal nuclei (neurons); GFAP, glial fibrillary acidic protein (astrocytes); Iba1, ionized binding adaptor molecule 1 (microglia); PLP, proteolipid protein (oligodendrocytic myelin).

In cases where our antibodies produced no labeling or only isolated labeled cells, we repeated the IHC procedures following the application of an antigen retrieval protocol. Antigenicity was recovered and produced a homogeneous labeling for all four markers in SSS and AFS-fixed brains (Figure 6). Nonetheless, NeuN and GFAP antigenicity was not recovered in three NBF fixed cases.

### 3.4 Histochemical stains

**Figure 7:**
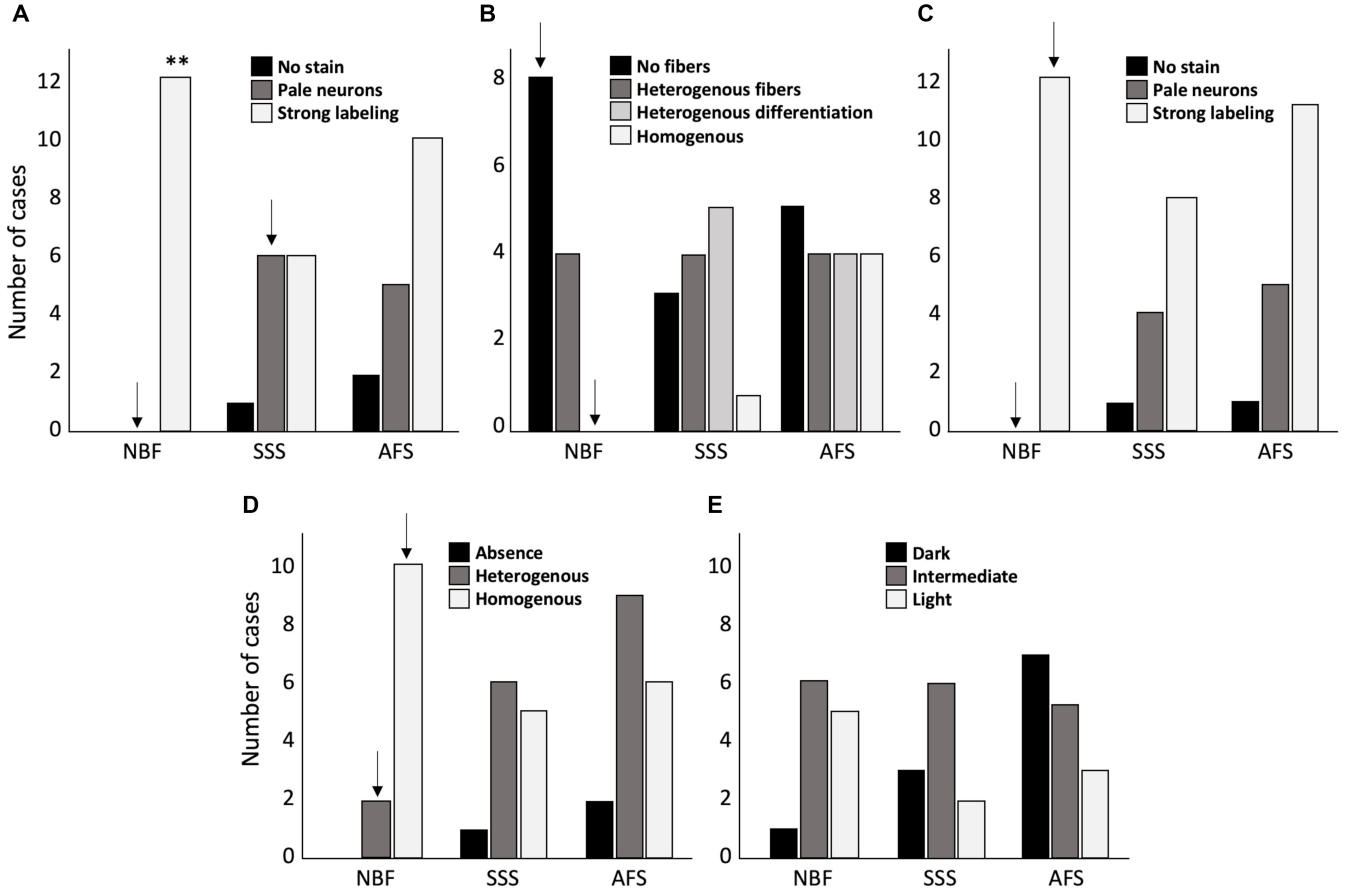
Bar charts of the quality of the histochemical stains. **(A)** Cresyl violet staining quality assessed by the absence of labeling = 0 (black), pale neurons = 1 (dark gray) or strong labeling = 2 (white). **(B)** Luxol fast blue staining quality, assessed by the absence of fibers = 0 (black), heterogeneous fibers = 1 (dark gray), heterogeneous differentiation = 2 (light gray) and homogeneous staining = 3 (white). **(C)** Prussian blue staining quality assessed by the absence of labeling = 0 (black), pale neurons = 1 (dark gray) or strong labeling = 2 (white). **(D)** Fibers preservation in Bielchowsky staining assessed by absence of fibers = 0 (black), heterogeneous distribution of the fibers = 1 (dark gray), or homogeneous distribution = 2 (white). **(E)** Bielchowsky’s staining background which could be dark = 0 (black), intermediate = 1 (dark gray) or light = 2 (white). **, Significant after Bonferroni correction (*p* < 0.01); ↓, Significant before Bonferroni correction; NBF, neutral-buffered formalin; SSS, salt-saturated solution; AFS, alcohol-formaldehyde solution.

First, we assessed quality of the Nissl-stain. We found a significantly higher frequency of cases showing high cellular contrast in NBF-fixed specimens (strong labeling of neurons in all the cases) (*p* = 0.0037), and less cases with pale neurons (*p* = 0.016; significant before a Bonferroni correction only). The brains fixed with the other two solutions sometimes produced pale neurons staining (SSS: *N* = 6, *p* = 0.045; significant before a Bonferroni correction only, and AFS: *N* = 5), or even no staining at all (SSS: *N* = 1; AFS: *N* = 2) (Figure 7A).

Second, we assessed the quality of myelinated fibers labeling with LFB. NBF-fixed brains showed a higher frequency of cases in which stained fibers were absent (score = 0) (*p* = 0.016; significant before a Bonferroni correction only) and never homogeneous distribution (*p* = 0.036; significant before a Bonferroni correction only). Fiber labeling with LFB in SSS and AFS-fixed brains was quite inconsistent as they showed randomly distributed scores (Figure 7B).

**Figure 8:**
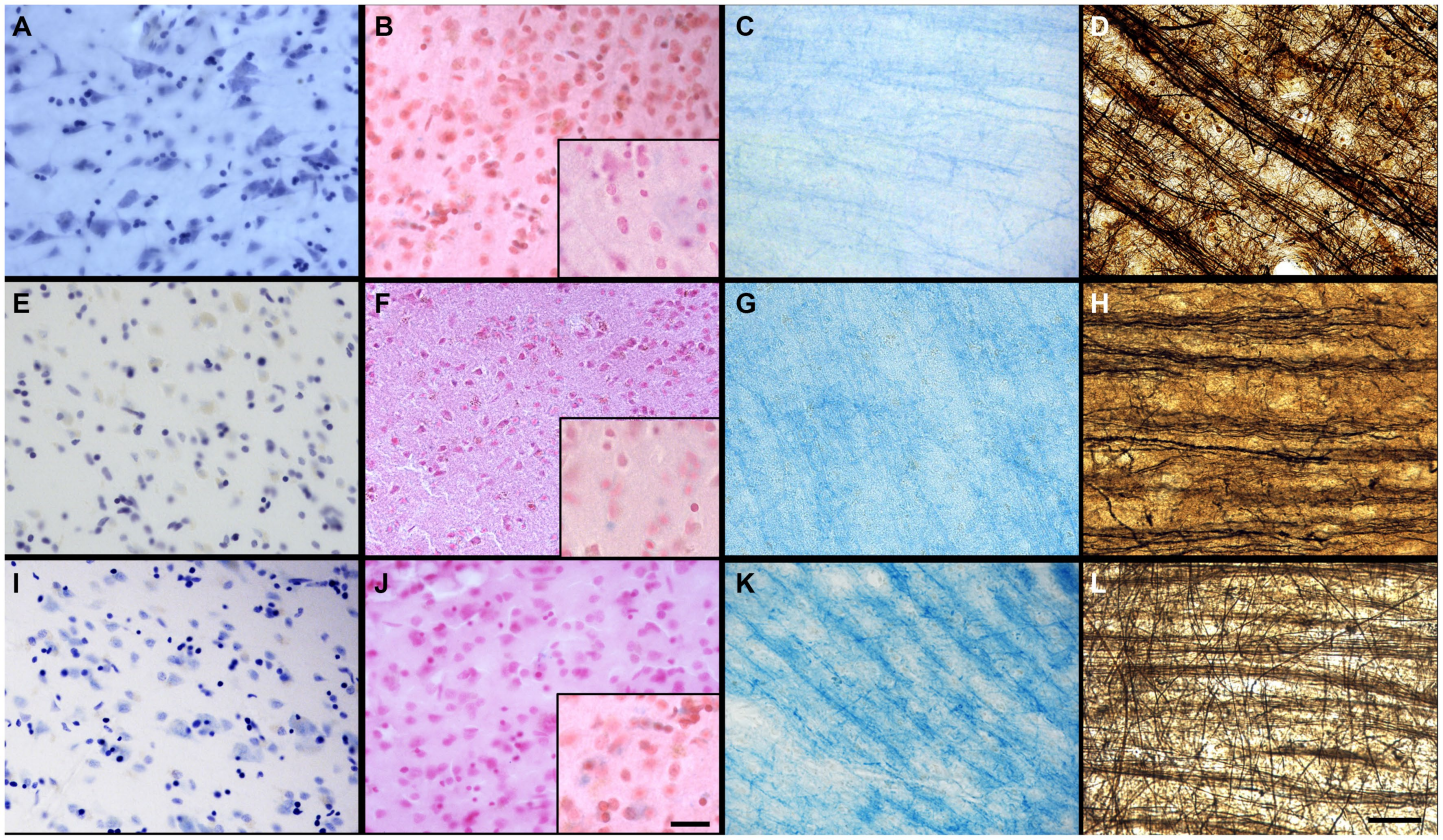
Quality of the histochemical stains. Photomicrographs (40X) of the Cresyl violet staining (neurons and glial cells in dark purple) for NBF **(A)**, SSS **(E)**, and AFS **(I)**, of the Prussian blue staining (neurons and glial cells in pink, iron in blue, with iron showed in inserts (100X) Scale bar = 50 μm) for NBF **(B)**, SSS **(F)**, and AFS **(J)**, of the LFB staining (myelin fibers in dark blue) for NBF **(C)**, SSS **(G)**, and AFS **(K)**, and Bielchowsky’s staining (axons and neurofibrils in dark brown) for NBF **(D)**, SSS **(H)**, and AFS **(L)**. Scale bar = 50 μm [valid for **(A–L)**].

Cell to background contrast was high following a Nuclear fast red (Nissl) counterstain to the Prussian blue staining (Figure 7C), regardless of the fixative. High cellular contrast occurred at a higher frequency in NBF specimens (all cases showed dark neurons: score = 2) (*p* = 0.016; significant before a Bonferroni correction only). Also, despite that not all the specimens showed ferric iron aggregation (depending on the pathology or age of the patient), blue spots indicating iron deposits were present in 10 NBF, 6 SSS and 8 AFS-fixed brains demonstrating the likelihood of detecting these deposits when either of these fixation protocols are applied (Figure 8, inserts in B, F, J).

Finally, we found a higher frequency of cases showing homogeneous labeling of silver impregnation of axonal fibers with the Bielchowsky procedure in the NBF-fixed brains (*p* = 0.0093; significant before a Bonferroni correction only) (Figure 7D). However, a higher occurrence of dark or intermediate background staining, was observed in the SSS and AFS compared to the NBF fixed brains, but this difference was not statistically significant (Figure 7E).

### 3.5 Confounding variables

#### 3.5.1 Post-mortem interval

More than half of the specimens were harvested following a short PMI (25 of 42). Longer PMI occurred more often in the NBF-fixed group than the other two groups (8 of 12; *p* = 0.028; significant before a Bonferroni correction only) (See Supplementary Table S1 and Supplementary Figures S1, S2 for details). Also, when treating PMI as a continuous variable and comparing the median values across groups (NBF = 39.25; SSS = 24; AFS = 22), NBF PMIs, although considerably longer, did not reach significance using Kruskal-Wallis (*p* = 0.084). However, given the significant difference obtained using Chi-square, we investigated the variables of interest separately in each of these groups.

In brains fixed with NBF, we found that specimens with shorter PMI were very easy to manipulate while specimens with longer PMI only scored good or poor (before Bonferroni correction, *p* = 0.028). There were no statistically significant differences in tissue quality (i.e., neuropil uniformity and cellular shape) in any of the fixative groups.

The effect of the PMI on the preservation of antigenicity was assessed separately for the four antibodies. For NeuN, we found unlabeled cells more often in NBF-fixed brains with longer PMI, and homogeneous patchy distribution of cells in the cases with a shorter PMI (before Bonferroni correction, *p* = 0.028). We found eight AFS cases with homogeneous GFAP distribution in the short PMI group, compared to none in the longer PMI group (statistically significant before Bonferroni correction, *p* = 0.012). We found no statistical difference of Iba1 labeling between the two groups (short or long PMI), in each fixative groups. In the NBF-fixed brains, there were more cases with no PLP labeling in the short PMI group (*N* = 2), while no such cases were observed in the group with a longer PMI (significant only before Bonferroni correction, *p* = 0.028).

We also assessed the quality of histochemical stains of the specimens in relation to the shorter or longer PMI. We found no statistical differences of the quality of Cresyl Violet, Luxol fast blue, Prussian blue stains and the two variables of interest for Bielchowsky staining, in each of the fixative groups.

#### 3.5.2 Histology processing delay

The occurrence of short and long HDs was not statistically significant between the fixative groups; 20 specimens showed shorter and 22 showed longer delays. Within the AFS-fixed brains, we obtained more cases with a longer HD (11 of 17) but this was not statistically significant using a Chi-square test. However, when treated as a continuous variable, and assessing the differences using Kruskal-Wallis, the median HD for AFS brains was significantly longer than for the other groups (NBF = 3.5; SSS = 2; AFS = 6; *p* = 0.008).

We found no statistical difference neither for the ease of manipulation nor for the neuropil uniformity of sections between cases with a shorter or longer histology delay (HD), in each fixative group. There was no difference of the cellular shape scores between cases with shorter or longer HD for SSS and NBF-fixed cases. For AFS, although not statistically significant, we observed a higher occurrence of irregular (shrunken and shriveled) cells in cases with longer HD (*N* = 7), while only one case with irregular cells was observed with shorter HD.

Regarding NeuN distribution, we found no differences between the short and long HD in each fixative group. We found more homogeneous distribution of GFAP labeling in the AFS-fixed brains with shorter HD (before Bonferroni correction, *p* = 0.028), and a patchier distribution of labeled cells with a longer HD (before Bonferroni correction, *p* = 0.012). Furthermore, when comparing between HD groups in SSS and NBF groups, homogeneous distribution of GFAP labeling was never found in cases with longer HD. For Iba1 and PLP, we found no statistical difference in each fixative group.

We also assessed the quality of histochemical procedures in each group. We found no statistical differences in the Luxol fast blue staining quality. However, we found a higher frequency of unlabeled and faintly labeled cells by Cresyl violet and the Nuclear red counterstaining of Prussian blue, in AFS-fixed brains with a long HD, but these differences did not reach statistical significance. When assessing the fibers preservation in Bielchowsky staining in each fixative group, we found that SSS-fixed brains with longer HD showed absence or heterogeneous fiber labeling, but this did not reach statistical significance. In the AFS-fixed brains, no labeled fibers were observed in the shorter HD group (*p* = 0.046; significant before a Bonferroni correction only). Regarding the Bielchowsky’s background, we found a higher frequency of cases with light backgrounds in the NBF-fixed brains with shorter HD and more cases with intermediate backgrounds with longer HD (before Bonferroni correction, *p* = 0.021). We also found more cases with dark Bielchowsky backgrounds with longer HD in AFS-fixed brains, but this was not statistically significant.

## 4 Discussion

### 4.1 Ease of manipulation

We were expecting that the SSS and AFS-fixed brains would be difficult to manipulate (softer tissue), according to our previous study in mice (Frigon et al., 2022). Accordingly, recent study in rat brains (Martins-Costa et al., 2022), and human anatomical studies describe these fixatives as soft-embalming solutions, which could lead to differences in the texture of the brains (Hopwood et al., 1989; Eltoum et al., 2001; Nicholson et al., 2005; Hammer et al., 2012; Hayashi et al., 2014; Balta et al., 2015; Tomalty et al., 2019; Rahman et al., 2021). The human tissue samples were relatively stiff and well-fixed macroscopically, and we found mostly that sections from AFS fixed specimens were easy to manipulate, and slightly more difficult in cases fixed with SSS. Nevertheless, SSS-fixed human sections were relatively easier to manipulate (stiffer) than the mouse sections of brains fixed with the same solutions (Frigon et al., 2022). This could be related to their different capacity to absorb the fixative (diffusion), related to their differences in shape, size, gyrification, and vascularization circuits across species (Ventura-Antunes et al., 2013).

### 4.2 Tissue quality

Integrity of the neuropil was compromised in the three groups in which we found a higher occurrence of fissures. Conversely, a more uniform neuropil was observed in NBF-fixed mouse brain sections (Frigon et al., 2022). This could be explained by the quick perfusion fixation of mice and timely histological processing soon after fixation compared to the lengthy months to years delay before histological processing of the brain bank NBF-fixed human specimens which may enhance chemical artefacts on the tissue. Regarding the broken neuropils, the high concentration of isopropyl alcohol or ethanol in SSS- and AFS-fixed brains, likely dehydrates tissues significantly (Viktorov and Proshin, 2003), and may cause fissures in the neuropil by shrinking, by changing the osmolarity of the intracellular vs. extracellular spaces. This may also explain the irregular aspect of the cells in the SSS and AFS-fixed brains, compared to the NBF-fixed brains that only show regular cell contours.

### 4.3 Antigenicity preservation

We found that NeuN and GFAP antigenicity was not well preserved in all cases, especially in NBF-fixed brains, where no cells were observed in three specimens. This was expected of the NBF-fixed brain bank specimens, which are preserved in a solution with a higher concentration of formaldehyde often for years before histology processing (i.e., longer histology delays). The cross-linking, due to the over-fixation by formaldehyde immersion (Arnold et al., 1996; Stumptner et al., 2019) likely affected the conformation of the neuronal nuclei and the glial fibrillary acidic protein more profoundly than the other two analyzed antigens, due to a higher interaction between their specific tertiary conformations and the chemicals, which is in line with the qualitative description of formaldehyde and its chemical reactions (Fox et al., 1985; Puchtler and Meloan, 1985; Helander, 1994; Kiernan, 2000; Thavarajah et al., 2012; Hade et al., 2024). Moreover, an antigen retrieval protocol recovered the antigenicity for Iba1 and PLP in all cases in a homogeneous way, whereas NeuN and GFAP did not regain antigenicity in some NBF-fixed brains.

Antigenicity was generally best preserved by the AFS fixative. SSS fixation produced mostly labeling of isolated cells or patches. However, an antigen retrieval protocol recovered all the antigens in tissue fixed with the two solutions used in anatomy laboratories, either SSS or AFS. Therefore, sufficient quality IHC procedures are possible following the application of an antigen retrieval protocol. These observations differ from what we reported in our previous study in mice, in which the antigenicity of the four main cell populations was preserved and homogeneously distributed across all the types of fixation (Frigon et al., 2022), even when no antigen retrieval protocol was applied prior to the IHC procedures. This also supports the differences in mice and human brain tissue preservation properties.

### 4.4 Histochemical stains

The clear labeling of cells with Cresyl violet and the Nuclear red counterstain of the Prussian blue procedure, demonstrates that the brains fixed with solutions from anatomy laboratories could be used for cytoarchitectonic analysis (Amunts and Zilles, 2015; Amunts et al., 2020; García-Cabezas et al., 2020), brain morphology (Zachlod et al., 2022), neurodegenerative disease (Bobinski et al., 2000), and MRI validation (Hoffmann et al., 2011; Magnain et al., 2014; Alkemade et al., 2020). Furthermore, Prussian blue (Perl’s stain) was feasible in specimens fixed with the three solution and helped detecting ferric iron deposits.

Luxol fast blue fiber staining was not homogeneous in NBF fixed brain sections. In some NBF fixed cases, labeling luxol fast blue failed to stain myelinated fibers altogether. Similarly, luxol fast blue staining of myelinated fibers was not consistent in both SSS and AFS-fixed brains suggesting that IHC (i.e., PLP in our case) might be a better approach to routinely label the myelinated axons. Indeed, IHC (i.e., MBP, MAG, PLP antigens) are widely used in neuropathology (Stüber et al., 2014; Kuhlmann et al., 2017; Adiele and Adiele, 2019; Vakrakou et al., 2022; Beach et al., 2023).

Bielchowsky silver impregnation of fibers, was best achieved in NBF-fixed brains, followed by SSS and AFS in which more cases with heterogeneous staining and intermediate density backgrounds were observed. This may be related to the ethanol and/or amount of alcohols in their chemical compositions. An old study on silver impregnation technique showed that lipid extraction with organic solvents such as ethanol can lead to unsatisfactory impregnation and poor staining (Wolman, 1958). However, we found good cases and high-quality stains following all fixation procedures, suggesting that the fixatives used in anatomy labs may be reliable for neuroscientific research.

### 4.5 Confounding variables

It is known that molecular conformations change at different paces after death, depending on the molecules being analyzed and the environmental conditions that affect the bodies (e.g., high temperatures). The changes happen within minutes for some cases, and within the first few hours of PMI for others. We therefore expected better histological results in brains with shorter PMI (Spokes, 1979; Palmer et al., 1988; Ravid et al., 1992; Hynd et al., 2003; Beach et al., 2015) and with a short HD, avoiding excessive post-mortem tissue decay and cross-linking from over-fixation (Arnold et al., 1996; Schulz et al., 2011; Stumptner et al., 2019). However, in our convenience sample, we found no evidence that neither lengthy PMI nor HD significantly affected the tissue; some good quality histology was obtained even in the cases with longer delays. This is in line with the recent work of Hade and their team, which reports good quality histology results with PMIs up to 37 h (Hade et al., 2024). It is worth noting that some individual cases suggested a slight influence of the PMI in the NBF-fixed brains, on the ease of manipulation, and antigenicity preservation of NeuN. These NBF brain bank tissue samples had significantly greater PMI (median = 45 h) than the anatomy laboratory specimens (median = 22 h) (*p* < 0.001), so it is conceivable that brains fixed with longer delays in anatomy labs could show the same patterns than brain bank specimens. However, we could not test this, given that our laboratory generally refuses bodies with PMIs of 48 h or more. Finally, we found significantly more homogeneous GFAP distribution in cases with a shorter HD and PMI in AFS-fixed brains, suggesting that this antigen might be more affected by these confounding variables, as reported by a recent study (Hade et al., 2024). Other results were not impacted in AFS-fixed brains, in which all histological procedures were successful.

Our results suggest that overall PMI and HD do not seem to impact the tissue quality. However, we saw a bit of an impact in some specific variables, such as ease of manipulation, NeuN and GFAP antigenicity. So, we consider that the prioritization of shorter delays should be preferred when feasible. Future studies increasing the number of specimens will elucidate more precisely the impact of longer delays on specific antigens.

### 4.6 Anatomy laboratory’s fixatives

#### 4.6.1 Salt saturated solution

We suggest that the SSS-fixed brains could be appropriate for neuroscientific research, since all the procedures were successful. However, these tissue blocks were more difficult to manipulate, and the overall quality of the tissue was poorer, thus SSS-fixed brains require more careful manipulation. Furthermore, antigenicity preservation was inadequate, but we demonstrated that IHC is still feasible following the application of an antigen retrieval protocol. We speculate that the lighter fixation of SSS brains owing to the low formaldehyde concentration led to a poor tissue preservation. The high osmolarity of this solution might also impact the diffusion of the fixative through the capillaries and interstitial compartments of the brain. Indeed, the SSS is over saturated in salt, which could lead to absorption of the free water in the brain prior to fixation with phenol and the small amount of formaldehyde contained in the solution (Coleman and Kogan, 1998; Hayashi et al., 2014), generating a lighter fixation. In addition, the cryoprotection in sucrose was very long (i.e., over a week compared to 1–2 days for the blocks fixed with the other two solutions) for these specimens; this might have also affected the tissue quality creating freezing artifacts.

#### 4.6.2 Alcohol-formaldehyde solution

Our results show that the AFS-fixed brains provided the best quality histology. Tissue was manipulated with ease, was of good quality, and produced the best IHC antigenicity preservation and quality of histochemical stains. Here again, an antigen retrieval protocol prior to the IHC procedures was warranted for optimal results in some specimens. The brains from the AFS group were also, for the most part, the oldest specimens (with a range of HD between 1 and 15 years), but even the old specimens held up well. AFS contains a higher concentration of formaldehyde (1.4%), compared to SSS (0.3% formaldehyde), which probably improved the fixation level of the brain. On the other hand, the lower formaldehyde concentration in the AFS compared to NBF, might reduce excessive cross-linking, making for the better preservation of antigenicity. Therefore, our results suggest that fixation with approximately 1% formaldehyde might be ideal and should be tested in future studies.

It is known that alcohol solutions might be optimal for combining gross anatomy dissection, surgical training and histology assessment of different tissues (Hopwood et al., 1989; O'Sullivan and Mitchell, 1993; Barton et al., 2009; Benet et al., 2014; Tomalty et al., 2019; Venne et al., 2020; Rahman et al., 2021; Martins-Costa et al., 2022). In this work, we have extensively assessed our specific solution, as it differed, although slightly, from the other formulas that are used worldwide (Hopwood et al., 1989; O'Sullivan and Mitchell, 1993; Barton et al., 2009; Benet et al., 2014; Cabrera et al., 2017; Tomalty et al., 2019; Shetty et al., 2020; Venne et al., 2020; Rahman et al., 2021; Martins-Costa et al., 2022). To our knowledge, no brain histology quality and antigenicity preservation assessments are available in the literature for such solutions. Altogether, our results suggest that AFS-fixed brains are of sufficient quality for qualitative or/and quantitative neuroscientific research.

#### 4.6.3 Neutral-buffered formalin

As explained before, 10% NBF is currently the most common fixative used in brain research but seems to be not ideal for the optimal preservation of specific antigens (e.g., GFAP). Also, NBF is generally used in brain banks that use immersion-fixation procedures, which may have a negative impact on the over-fixation and cross-linking. At the same time, gross anatomy laboratories that use chemical fixation through perfusion of the whole-body cannot use this high concentration of NBF, since the bodies are typically used for dissection which would determine a long-term exposure to hazards for the users. However, a recent study of Insausti et al. provided very good histology results following a carotid-perfusion method that enables to deliver formaldehyde to the brain, while allowing the injection of another fixative for the rest of the body (Insausti et al., 2023). This could be an ideal solution in pursuing the utilization of 10% NBF for brain fixation by perfusion (avoiding the immersion confounds), while avoiding the hazard and inconvenience of high concentrations of formaldehyde for the dissection.

### 4.7 Study limitations

First, working with human post-mortem tissue involves dealing with confounding variables, such as the post-mortem delay and the histology delay. The brains could also be affected by other variables such as the age, sex, presence of comorbidities and fixative perfusion quality (i.e., embalming of the full body). The post-mortem and histology delays have been assessed as confounding variables in each fixative group separately, and we found that they did not significantly impact our results. However, we acknowledge that the very different PMI and HD across fixative groups may affect the variables of interest, so further investigations across solutions in balanced samples with similar ranges of PMI and HD are warranted. Additionally, although our specimens were from a convenience sample, they mostly included normal-aging brains with absence of neuronal or vascular comorbidities, with a random distribution of sex. Also, all the brain specimens were removed from adequately fixed whole bodies that were used for regular dissection courses, translating an overall adequate perfusion procedure. However, a more detailed assessment of the perfusion of the fixative during the embalming (e.g., quantification of thrombi removed from the carotid arteries and jugular veins) could be used as an additional variable of interest in future studies. Second, using a convenience sample ensured that we obtained tissues from different brain lobes. However, we confirm that we used neocortex for all the specimens. Third, free-floating tissue sections stained with IHC procedures might show heterogeneous labeling, since the staining depends on the level of antibody penetration and the tissue background. Analyses might therefore be affected by the protocol itself, which was also evaluated as a variable of interest in our previous study in mice brains (Frigon et al., 2022). Therefore, the protocols were already tested and validated for this study, so we excluded the antibody penetration and tissue background quality as variables here. Fourth, we are aware that other antigens of interest could have been tested, but we limited our study to the antigens that were previously validated in our study in mice (Frigon et al., 2022). Fifth, we acknowledge that our study was conducted in a qualitative manner. However, quantitative assessment of the four cell types would be necessary to determine whether results obtained across solutions would be valid to assess specific pathological changes of the brains (e.g., loss of neurons in an area due to neurodegeneration in a brain fixed with NBF, vs. a brain fixed with SSS or AFS). Finally, the statistical power might have been limited by our relatively small sample size, especially when dealing with confounding variables. However, availability of human brain tissue is limited, and our study included all the specimens that were available at the time of the assessments. Future studies with larger sample sizes are warranted to further our understanding of the impact of confounding factors.

## 5 Conclusion

In this study, we compared the quality of histochemical and immunohistochemical procedures performed on brain tissue from cases fixed by current embalming protocols in use in gross anatomy dissection laboratories and fixation protocols in use in major brain bank settings. We showed lower overall histology quality using a salt saturated solution but also a higher quality for some specific variables when using an alcohol-formaldehyde solution in comparison to the classic neutral-buffered formalin solution used in brain banks. We further showed that immunohistochemistry and histochemical stains are feasible with brains fixed with two solutions used in gross anatomy laboratories following the application of a heat-induced antigen retrieval protocol. Therefore, brains coming from body donation programs for teaching gross-anatomy could also be used for neuroscientific research, which could increase the amount of brain tissue available to neuroscientists.

## Data availability statement

The original contributions presented in the study are included in the article/Supplementary material, further inquiries can be directed to the corresponding author.

## Ethics statement

The studies involving humans were approved by Ethics Subcommittee of the Anatomy Teaching and Research Laboratory (SCÉLERA) of the Université du Québec à Trois-Rivières. The studies were conducted in accordance with the local legislation and institutional requirements. The participants provided their written informed consent to participate in this study.

## Author contributions

EM-F: Writing – original draft, Writing – review & editing, Conceptualization, Data curation, Formal analysis, Investigation, Methodology, Visualization. AG-L: Data curation, Methodology, Writing – review & editing. MD: Data curation, Formal analysis, Methodology, Resources, Writing – review & editing. DB: Conceptualization, Data curation, Formal analysis, Funding acquisition, Methodology, Project administration, Resources, Supervision, Validation, Writing – review & editing. JM: Conceptualization, Data curation, Formal analysis, Funding acquisition, Investigation, Methodology, Project administration, Resources, Supervision, Writing – original draft, Writing – review & editing.

## Footnotes

^1^ https://pathologycenter.jp/method-e/

^2^ https://www.abcam.com/products/assay-kits/iron-stain-kit-prussian-blue-stain-ab150674.html#

^3^ https://www.ihcworld.com/_protocols/special_stains/bielschowsky.htm

## References

[ref1] Adiele R. C. Adiele C. A. (2019). Metabolic defects in multiple sclerosis. Mitochondrion 44, 7–14. doi: 10.1016/j.mito.2017.12.005 29246870

[ref2] Alkemade A. Pine K. Kirilina E. Keuken M. C. Mulder M. J. Balesar R.. (2020). 7 tesla MRI followed by histological 3D reconstructions in whole-brain specimens. Front. Neuroanat. 14:536838. doi: 10.3389/fnana.2020.536838, PMID: 33117133 PMC7574789

[ref3] Allsop D. (2000). Introduction to Alzheimer's disease. Methods Mol. Med. 32, 1–22. doi: 10.1385/1-59259-195-7:1 21318508

[ref4] Amunts K. Mohlberg H. Bludau S. Zilles K. (2020). Julich-brain: a 3D probabilistic atlas of the human brain's cytoarchitecture. Science 369, 988–992. doi: 10.1126/science.abb4588, PMID: 32732281

[ref5] Amunts K. Zilles K. (2015). Architectonic mapping of the human brain beyond Brodmann. Neuron 88, 1086–1107. doi: 10.1016/j.neuron.2015.12.001, PMID: 26687219

[ref6] Arnold M. M. Srivastava S. Fredenburgh J. Stockard C. R. Myers R. B. Grizzle W. E. (1996). Effects of fixation and tissue processing on immunohistochemical demonstration of specific antigens. Biotech. Histochem. 71, 224–230. doi: 10.3109/10520299609117164, PMID: 8896794

[ref7] Balta J. Y. Cronin M. Cryan J. F. O'Mahony S. M. (2015). Human preservation techniques in anatomy: a 21st century medical education perspective. Clin. Anat. 28, 725–734. doi: 10.1002/ca.22585, PMID: 26118424

[ref8] Barton D. P. Davies D. C. Mahadevan V. Dennis L. Adib T. Mudan S.. (2009). Dissection of soft-preserved cadavers in the training of gynaecological oncologists: report of the first UK workshop. Gynecol. Oncol. 113, 352–356. doi: 10.1016/j.ygyno.2009.02.012, PMID: 19282022

[ref9] Beach T. G. Adler C. H. Sue L. I. Serrano G. Shill H. A. Walker D. G.. (2015). Arizona study of aging and neurodegenerative disorders and brain and body donation program. Neuropathology 35, 354–389. doi: 10.1111/neup.12189, PMID: 25619230 PMC4593391

[ref10] Beach T. G. Sue L. I. Scott S. Intorcia A. J. Walker J. E. Arce R. A.. (2023). Cerebral white matter rarefaction has both neurodegenerative and vascular causes and may primarily be a distal axonopathy. J. Neuropathol. Exp. Neurol. 82, 457–466. doi: 10.1093/jnen/nlad026, PMID: 37071794 PMC10209646

[ref11] Beach T. G. Tago H. Nagai T. Kimura H. McGeer P. L. McGeer E. G. (1987). Perfusion-fixation of the human brain for immunohistochemistry: comparison with immersion-fixation. J. Neurosci. Methods 19, 183–192. doi: 10.1016/s0165-0270(87)80001-8, PMID: 2437408

[ref12] Benet A. Rincon-Torroella J. Lawton M. T. González Sánchez J. J. (2014). Novel embalming solution for neurosurgical simulation in cadavers. J. Neurosurg. 120, 1229–1237. doi: 10.3171/2014.1.Jns131857, PMID: 24527814

[ref13] Birkl C. Langkammer C. Golob-Schwarzl N. Leoni M. Haybaeck J. Goessler W.. (2016). Effects of formalin fixation and temperature on MR relaxation times in the human brain. NMR Biomed. 29, 458–465. doi: 10.1002/nbm.3477, PMID: 26835664

[ref14] Bobinski M. de Leon M. J. Wegiel J. Desanti S. Convit A. Saint Louis L. A.. (2000). The histological validation of post mortem magnetic resonance imaging-determined hippocampal volume in Alzheimer's disease. Neuroscience 95, 721–725. doi: 10.1016/s0306-4522(99)00476-5, PMID: 10670438

[ref15] Brenner E. (2014). Human body preservation - old and new techniques. J. Anat. 224, 316–344. doi: 10.1111/joa.12160, PMID: 24438435 PMC3931544

[ref16] Bronge L. Bogdanovic N. Wahlund L. O. (2002). Postmortem MRI and histopathology of white matter changes in Alzheimer brains. A quantitative, comparative study. Dement. Geriatr. Cogn. Disord. 13, 205–212. doi: 10.1159/000057698 12006730

[ref17] Cabrera N. C. Espinoza J. R. Vargas-Jentzsch P. Sandoval P. Ramos L. A. Aponte P. M. (2017). Alcohol-based solutions for bovine testicular tissue fixation. J. Vet. Diagn. Invest. 29, 91–99. doi: 10.1177/1040638716672252, PMID: 27852815

[ref18] Cardoso J. R. Pereira L. M. Iversen M. D. Ramos A. L. (2014). What is gold standard and what is ground truth? Dental Press J. Orthod. 19, 27–30. doi: 10.1590/2176-9451.19.5.027-030.ebo, PMID: 25715714 PMC4296658

[ref19] Carlos A. F. Poloni T. E. Medici V. Chikhladze M. Guaita A. Ceroni M. (2019). From brain collections to modern brain banks: a historical perspective. Alzheimers Dement (N Y) 5, 52–60. doi: 10.1016/j.trci.2018.12.002, PMID: 30775417 PMC6365388

[ref20] Churukian C. J. (2008). “14 - pigments and minerals” in Theory and practice of histological techniques. eds. Bancroft J. D. Gamble M.. 6th ed (London: Churchill Livingstone), 233–259.

[ref21] Coleman R. Kogan I. (1998). An improved low-formaldehyde embalming fluid to preserve cadavers for anatomy teaching. J. Anat. 192, 443–446. doi: 10.1046/j.1469-7580.1998.19230443.x, PMID: 9688512 PMC1467790

[ref22] Connor J. R. Benkovic S. A. (1992). Iron regulation in the brain: histochemical, biochemical, and molecular considerations. Ann. Neurol. 32, S51–S61. doi: 10.1002/ana.410320710, PMID: 1510381

[ref23] Dawe R. J. Bennett D. A. Schneider J. A. Vasireddi S. K. Arfanakis K. (2009). Postmortem MRI of human brain hemispheres: T2 relaxation times during formaldehyde fixation. Magn. Reson. Med. 61, 810–818. doi: 10.1002/mrm.21909, PMID: 19189294 PMC2713761

[ref24] den Bakker M. A. (2017). Is histopathology still the gold standard? Ned. Tijdschr. Geneeskd. 160:D981. 28120732

[ref25] Durand-Martel P. Tremblay D. Brodeur C. Paquet N. (2010). Autopsy as gold standard in FDG-PET studies in dementia. Can. J. Neurol. Sci. 37, 336–342. doi: 10.1017/s0317167100010222, PMID: 20481267

[ref26] Dusek P. Hofer T. Alexander J. Roos P. M. Aaseth J. O. (2022). Cerebral Iron deposition in neurodegeneration. Biomol. Ther. 12:714. doi: 10.3390/biom12050714, PMID: 35625641 PMC9138489

[ref27] Eilam-Altstädter R. Las L. Witter M. P. Ulanovsky N. (2021). “Methods” in Stereotaxic brain atlas of the Egyptian fruit bat. eds. Eilam-Altstädter R. Las L. Witter M. P. Ulanovsky N. (Cambridge, MA: Academic Press), 1–9.

[ref28] Elobeid A. Rantakömi S. Soininen H. Alafuzoff I. (2014). Alzheimer's disease-related plaques in nondemented subjects. Alzheimers Dement. 10, 522–529. doi: 10.1016/j.jalz.2012.12.009, PMID: 24742915

[ref29] Eltoum I. Fredenburgh J. Myers R. B. Grizzle W. E. (2001). Introduction to the theory and practice of fixation of tissues. J. Histotechnol. 24, 173–190. doi: 10.1179/his.2001.24.3.173

[ref30] Fan S. Zheng Y. Liu X. Fang W. Chen X. Liao W.. (2018). Curcumin-loaded PLGA-PEG nanoparticles conjugated with B6 peptide for potential use in Alzheimer's disease. Drug Deliv. 25, 1091–1102. doi: 10.1080/10717544.2018.1461955, PMID: 30107760 PMC6116673

[ref31] Fisher M. M. (1905). The toxic effects of formaldehyde and formalin. J. Exp. Med. 6, 487–518. doi: 10.1084/jem.6.4-6.487, PMID: 19866982 PMC2124506

[ref32] Fox C. H. Johnson F. B. Whiting J. Roller P. P. (1985). Formaldehyde fixation. J. Histochem. Cytochem. 33, 845–853. doi: 10.1177/33.8.3894502 3894502

[ref33] Frigon E.-M. Dadar M. Boire D. Maranzano J. (2022). Antigenicity is preserved with fixative solutions used in human gross anatomy: a mice brain immunohistochemistry study. Front. Neuroanat. 16:957358. doi: 10.3389/fnana.2022.957358, PMID: 36312297 PMC9596787

[ref34] Frølich K. W. Andersen L. M. Knutsen A. Flood P. R. (1984). Phenoxyethanol as a nontoxic substitute for formaldehyde in long-term preservation of human anatomical specimens for dissection and demonstration purposes. Anat. Rec. 208, 271–278. doi: 10.1002/ar.1092080214, PMID: 6703343

[ref35] García-Cabezas M. Á. Hacker J. L. Zikopoulos B. (2020). A protocol for cortical type analysis of the human neocortex applied on histological samples, the atlas of Von Economo and Koskinas, and magnetic resonance imaging. Front. Neuroanat. 14:576015. doi: 10.3389/fnana.2020.576015, PMID: 33364924 PMC7750391

[ref36] Gouw A. A. Seewann A. van der Flier W. M. Barkhof F. Rozemuller A. M. Scheltens P.. (2011). Heterogeneity of small vessel disease: a systematic review of MRI and histopathology correlations. J. Neurol. Neurosurg. Psychiatry 82, 126–135. doi: 10.1136/jnnp.2009.204685, PMID: 20935330

[ref37] Guindi M. (2018). “11 - liver disease in Iron overload” in Practical hepatic pathology: A diagnostic approach. ed. Saxena R.. 2nd ed (Amsterdam: Elsevier), 151–165.

[ref38] Gulati R. Ahuja M. S. Langer V. Gopal P. Rustagi S. M. Dave V. (2020). Comparative study of formalin solution and saturated salt solution for embalming cadavers for surgical skills training. Indian J. Anat. 9, 33–37. doi: 10.21088/ija.2320.0022.9120.5

[ref39] Hade A. C. Philips M. A. Promet L. Jagomäe T. Hanumantharaju A. Salumäe L.. (2024). A cost-effective and efficient ex vivo, ex situ human whole brain perfusion protocol for immunohistochemistry. J. Neurosci. Methods 404:110059. doi: 10.1016/j.jneumeth.2024.110059, PMID: 38218387

[ref40] Hammer N. Löffler S. Feja C. Sandrock M. Schmidt W. Bechmann I.. (2012). Ethanol-glycerin fixation with thymol conservation: a potential alternative to formaldehyde and phenol embalming. Anat. Sci. Educ. 5, 225–233. doi: 10.1002/ase.1270, PMID: 22434588

[ref41] Hayashi S. Homma H. Naito M. Oda J. Nishiyama T. Kawamoto A.. (2014). Saturated salt solution method: a useful cadaver embalming for surgical skills training. Medicine 93:e196. doi: 10.1097/md.0000000000000196 25501070 PMC4602773

[ref42] Hayashi S. Naito M. Kawata S. Qu N. Hatayama N. Hirai S.. (2016). History and future of human cadaver preservation for surgical training: from formalin to saturated salt solution method. Anat. Sci. Int. 91, 1–7. doi: 10.1007/s12565-015-0299-5, PMID: 26670696

[ref43] Helander K. G. (1994). Kinetic studies of formaldehyde binding in tissue. Biotech. Histochem. 69, 177–179. doi: 10.3109/10520299409106282, PMID: 8068812

[ref44] Herculano-Houzel S. Ribeiro P. Campos L. Valotta da Silva A. Torres L. B. Catania K. C.. (2011). Updated neuronal scaling rules for the brains of Glires (rodents/lagomorphs). Brain Behav. Evol. 78, 302–314. doi: 10.1159/000330825, PMID: 21985803 PMC3237106

[ref45] Hoffmann A. Bredno J. Wendland M. F. Derugin N. Hom J. Schuster T.. (2011). Validation of in vivo magnetic resonance imaging blood-brain barrier permeability measurements by comparison with gold standard histology. Stroke 42, 2054–2060. doi: 10.1161/strokeaha.110.597997, PMID: 21636816 PMC3921072

[ref46] Hopwood D. Slidders W. Yeaman G. R. (1989). Tissue fixation with phenol-formaldehyde for routine histopathology. Histochem. J. 21, 228–234. doi: 10.1007/bf01747525, PMID: 2674069

[ref47] Humphreys C. A. Jansen M. A. Muñoz Maniega S. González-Castro V. Pernet C. Deary I. J.. (2019). A protocol for precise comparisons of small vessel disease lesions between ex vivo magnetic resonance imaging and histopathology. Int. J. Stroke 14, 310–320. doi: 10.1177/1747493018799962, PMID: 30196792 PMC6604680

[ref48] Hynd M. R. Lewohl J. M. Scott H. L. Dodd P. R. (2003). Biochemical and molecular studies using human autopsy brain tissue. J. Neurochem. 85, 543–562. doi: 10.1046/j.1471-4159.2003.01747.x 12694381

[ref49] Insausti R. Insausti A. M. Muñoz López M. Medina Lorenzo I. Arroyo-Jiménez M. D. M. Marcos Rabal M. P.. (2023). Ex vivo, in situ perfusion protocol for human brain fixation compatible with microscopy, MRI techniques, and anatomical studies [methods]. Front. Neuroanat. 17:1149674. doi: 10.3389/fnana.2023.1149674, PMID: 37034833 PMC10076536

[ref50] Kanawaku Y. Someya S. Kobayashi T. Hirakawa K. Shiotani S. Fukunaga T.. (2014). High-resolution 3D-MRI of postmortem brain specimens fixed by formalin and gadoteridol. Leg. Med. (Tokyo) 16, 218–221. doi: 10.1016/j.legalmed.2014.03.003, PMID: 24709037

[ref51] Kiernan J. A. (2000). Formaldehyde, formalin, paraformaldehyde and glutaraldehyde: what they are and what they do. Microscopy Today 8, 8–13. doi: 10.1017/S1551929500057060

[ref52] Kishore M. Pradeep M. Narne P. Jayalakshmi S. Panigrahi M. Patil A.. (2023). Regulation of Keap1-Nrf2 axis in temporal lobe epilepsy-hippocampal sclerosis patients may limit the seizure outcomes. Neurol. Sci. 44, 4441–4450. doi: 10.1007/s10072-023-06936-0, PMID: 37432566

[ref53] Kovacs G. G. Budka H. (2010). Current concepts of neuropathological diagnostics in practice: neurodegenerative diseases. Clin. Neuropathol. 29, 271–288. doi: 10.5414/npp29271, PMID: 20860890

[ref54] Kruer M. C. (2013). The neuropathology of neurodegeneration with brain iron accumulation. Int. Rev. Neurobiol. 110, 165–194. doi: 10.1016/b978-0-12-410502-7.00009-0, PMID: 24209439 PMC4429307

[ref55] Kuhlmann T. Ludwin S. Prat A. Antel J. Brück W. Lassmann H. (2017). An updated histological classification system for multiple sclerosis lesions. Acta Neuropathol. 133, 13–24. doi: 10.1007/s00401-016-1653-y, PMID: 27988845

[ref56] Laule C. Kozlowski P. Leung E. Li D. K. Mackay A. L. Moore G. R. (2008). Myelin water imaging of multiple sclerosis at 7 T: correlations with histopathology. NeuroImage 40, 1575–1580. doi: 10.1016/j.neuroimage.2007.12.008, PMID: 18321730

[ref57] Laule C. Moore G. R. W. (2018). Myelin water imaging to detect demyelination and remyelination and its validation in pathology. Brain Pathol. 28, 750–764. doi: 10.1111/bpa.12645, PMID: 30375119 PMC8028667

[ref58] LeVine S. M. (1997). Iron deposits in multiple sclerosis and Alzheimer's disease brains. Brain Res. 760, 298–303. doi: 10.1016/s0006-8993(97)00470-8, PMID: 9237552

[ref59] Lindberg M. R. Lamps L. W. (2018). “Central nervous system” in Diagnostic pathology: Normal histology. eds. Lindberg M. R. Lamps L. W.. 2nd ed (Amsterdam: Elsevier), 108–111.

[ref60] Litchfield S. Nagy Z. (2001). New temperature modification makes the Bielschowsky silver stain reproducible. Acta Neuropathol. 101, 17–21. doi: 10.1007/s004010000248, PMID: 11194935

[ref61] Lombardero M. Yllera M. M. Costa E. S. A. Oliveira M. J. Ferreira P. G. (2017). Saturated salt solution: a further step to a formaldehyde-free embalming method for veterinary gross anatomy. J. Anat. 231, 309–317. doi: 10.1111/joa.12634, PMID: 28542788 PMC5522894

[ref62] Loomba S. Straehle J. Gangadharan V. Heike N. Khalifa A. Motta A.. (2022). Connectomic comparison of mouse and human cortex. Science 377:eabo0924. doi: 10.1126/science.abo0924, PMID: 35737810

[ref63] Love S. Nicoll J. A. (1992). Comparison of modified Bielschowsky silver impregnation and anti-ubiquitin immunostaining of cortical and nigral Lewy bodies. Neuropathol. Appl. Neurobiol. 18, 585–592. doi: 10.1111/j.1365-2990.1992.tb00830.x, PMID: 1283205

[ref64] Luna L. G. Armed Forces Institute of P. (1968). Manual of histologic staining methods of the armed forces Institute of Pathology (3rd). Blakiston Division, McGraw-Hill New York.

[ref65] Magnain C. Augustinack J. C. Reuter M. Wachinger C. Frosch M. P. Ragan T.. (2014). Blockface histology with optical coherence tomography: a comparison with Nissl staining. NeuroImage 84, 524–533. doi: 10.1016/j.neuroimage.2013.08.072, PMID: 24041872 PMC4315235

[ref66] Maranzano J. Dadar M. Bertrand-Grenier A. Frigon E. M. Pellerin J. Plante S.. (2020). A novel ex vivo, in situ method to study the human brain through MRI and histology. J. Neurosci. Methods 345:108903. doi: 10.1016/j.jneumeth.2020.108903, PMID: 32777310

[ref67] Martins-Costa C. Nunes T. C. Anjos-Ramos L. D. (2022). Anatomo-comparative study of formaldehyde, alcohol, and saturated salt solution as fixatives in Wistar rat brains. Anat. Histol. Embryol. 51, 740–745. doi: 10.1111/ahe.12852, PMID: 35964229

[ref68] Mouritzen Dam A. (1979). Shrinkage of the brain during histological procedures with fixation in formaldehyde solutions of different concentrations. J. Hirnforsch. 20, 115–119. PMID: 556570

[ref69] Musiał A. Gryglewski R. W. Kielczewski S. Loukas M. Wajda J. (2016). Formalin use in anatomical and histological science in the 19th and 20th centuries. Folia Med. Cracov. 56, 31–40. PMID: 28275269

[ref70] Nazemorroaya A. Aghaeifar A. Shiozawa T. Hirt B. Schulz H. Scheffler K.. (2022). Developing formalin-based fixative agents for post mortem brain MRI at 9.4 T. Magn. Reson. Med. 87, 2481–2494. doi: 10.1002/mrm.29122, PMID: 34931721

[ref71] Nicholson H. D. Samalia L. Gould M. Hurst P. R. Woodroffe M. (2005). A comparison of different embalming fluids on the quality of histological preservation in human cadavers. Eur. J. Morphol. 42, 178–184. doi: 10.1080/09243860500473306, PMID: 16982474

[ref72] Ogawa Y. (1990). “Silver impregnation method for neurons using synthetic surfactants: a contribution to Golgi method” in Quantitative and qualitative microscopy. ed. Conn P. M. (Amsterdam: Elsevier)

[ref73] O'Sullivan E. Mitchell B. S. (1993). An improved composition for embalming fluid to preserve cadavers for anatomy teaching in the United Kingdom. J. Anat. 182, 295–297. PMID: 8376206 PMC1259842

[ref74] Ouellette R. Mangeat G. Polyak I. Warntjes M. Forslin Y. Bergendal Å.. (2020). Validation of rapid magnetic resonance myelin imaging in multiple sclerosis. Ann. Neurol. 87, 710–724. doi: 10.1002/ana.25705, PMID: 32057118

[ref75] Palmer A. M. Lowe S. L. Francis P. T. Bowen D. M. (1988). Are post-mortem biochemical studies of human brain worthwhile? Biochem. Soc. Trans. 16, 472–475. doi: 10.1042/bst0160472, PMID: 2905309

[ref76] Puchtler H. Meloan S. N. (1985). On the chemistry of formaldehyde fixation and its effects on immunohistochemical reactions. Histochemistry 82, 201–204. doi: 10.1007/bf00501395 3997553

[ref77] Rahman S. M. N. Alam T. Alam N. N. (2021). Preservation of histology by phenol-based fixative: Mini review of recent findings %J. Int. J. Morphol. 39, 50–56. doi: 10.4067/S0717-95022021000100050

[ref78] Raja D. S. Sultana B. (2012). Potential health hazards for students exposed to formaldehyde in the gross anatomy laboratory. J. Environ. Health 74, 36–40. PMID: 22329207

[ref79] Ravid R. Van Zwieten E. J. Swaab D. F. (1992). Brain banking and the human hypothalamus--factors to match for, pitfalls and potentials. Prog. Brain Res. 93, 83–95. doi: 10.1016/s0079-6123(08)64565-3 1480765

[ref80] Roseborough A. D. Langdon K. D. Hammond R. Cipriano L. E. Pasternak S. H. Whitehead S. N.. (2020). Post-mortem 7 tesla MRI detection of white matter hyperintensities: a multidisciplinary voxel-wise comparison of imaging and histological correlates. NeuroImage: Clinical 27:102340. doi: 10.1016/j.nicl.2020.102340, PMID: 32679554 PMC7364158

[ref81] Schramek G. G. Stoevesandt D. Reising A. Kielstein J. T. Hiss M. Kielstein H. (2013). Imaging in anatomy: a comparison of imaging techniques in embalmed human cadavers. BMC Med. Educ. 13:143. doi: 10.1186/1472-6920-13-143, PMID: 24156510 PMC4016606

[ref82] Schulz G. Crooijmans H. J. Germann M. Scheffler K. Müller-Gerbl M. Müller B. (2011). Three-dimensional strain fields in human brain resulting from formalin fixation. J. Neurosci. Methods 202, 17–27. doi: 10.1016/j.jneumeth.2011.08.031, PMID: 21889536

[ref83] Selvaraji S. Efthymios M. Foo R. S. Y. Fann D. Y. Lai M. K. P. Chen C. L. H.. (2022). Time-restricted feeding modulates the DNA methylation landscape, attenuates hallmark neuropathology and cognitive impairment in a mouse model of vascular dementia. Theranostics 12, 3007–3023. doi: 10.7150/thno.71815, PMID: 35547760 PMC9065201

[ref84] Shetty J. K. Babu H. F. Hosapatna Laxminarayana K. P. (2020). Histomorphological assessment of formalin versus nonformalin fixatives in diagnostic surgical pathology. J. Lab. Physicians 12, 271–275. doi: 10.1055/s-0040-1722546, PMID: 33390677 PMC7773438

[ref85] Spencer L. T. Bancroft J. D. (2008). “6 - Tissue processing,” in Theory and practice of histological techniques (sixth edition). eds. Bancroft J. D. Gamble M.. Churchill Livingstone: Edinburgh. p. 83–92.

[ref86] Spokes E. G. (1979). An analysis of factors influencing measurements of dopamine, noradrenaline, glutamate decarboxylase and choline acetylase in human post-mortem brain tissue. Brain 102, 333–346. doi: 10.1093/brain/102.2.333, PMID: 455043

[ref87] Stahnisch F. W. (2015). Max Bielschowsky (1869-1940). J. Neurol. 262, 792–794. doi: 10.1007/s00415-014-7544-z, PMID: 25346063 PMC4363476

[ref88] Stüber C. Morawski M. Schäfer A. Labadie C. Wähnert M. Leuze C.. (2014). Myelin and iron concentration in the human brain: a quantitative study of MRI contrast. NeuroImage 93, 95–106. doi: 10.1016/j.neuroimage.2014.02.026, PMID: 24607447

[ref89] Stumptner C. Pabst D. Loibner M. Viertler C. Zatloukal K. (2019). The impact of crosslinking and non-crosslinking fixatives on antigen retrieval and immunohistochemistry. New Biotechnol. 52, 69–83. doi: 10.1016/j.nbt.2019.05.003, PMID: 31082574

[ref90] Switzer R. C. (2000). Application of silver degeneration stains for neurotoxicity testing. Toxicol. Pathol. 28, 70–83. doi: 10.1177/019262330002800109 10668992

[ref91] Thavarajah R. Mudimbaimannar V. K. Elizabeth J. Rao U. K. Ranganathan K. (2012). Chemical and physical basics of routine formaldehyde fixation. J. Oral. Maxillofac. Pathol. 16, 400–405. doi: 10.4103/0973-029x.102496, PMID: 23248474 PMC3519217

[ref92] Tomalty D. Pang S. C. Ellis R. E. (2019). Preservation of neural tissue with a formaldehyde-free phenol-based embalming protocol. Clin. Anat. 32, 224–230. doi: 10.1002/ca.23290, PMID: 30281854

[ref93] Troiano N. W. Ciovacco W. A. Kacena M. A. (2009). The effects of fixation and dehydration on the histological quality of Undecalcified murine bone specimens embedded in Methylmethacrylate. J. Histotechnol. 32, 27–31. doi: 10.1179/his.2009.32.1.27, PMID: 20160920 PMC2770706

[ref94] Uchihara T. (2007). Silver diagnosis in neuropathology: principles, practice and revised interpretation. Acta Neuropathol. 113, 483–499. doi: 10.1007/s00401-007-0200-2, PMID: 17401570 PMC1868652

[ref95] Vakrakou A. G. Brinia M. E. Svolaki I. Argyrakos T. Stefanis L. Kilidireas C. (2022). Immunopathology of Tumefactive demyelinating lesions-from idiopathic to drug-related cases. Front. Neurol. 13:868525. doi: 10.3389/fneur.2022.868525, PMID: 35418930 PMC8997292

[ref96] van Essen H. F. Verdaasdonk M. A. Elshof S. M. de Weger R. A. van Diest P. J. (2010). Alcohol based tissue fixation as an alternative for formaldehyde: influence on immunohistochemistry. J. Clin. Pathol. 63, 1090–1094. doi: 10.1136/jcp.2010.079905 20978021

[ref97] Venne G. Zec M. L. Welte L. Noel G. (2020). Qualitative and quantitative comparison of Thiel and phenol-based soft-embalmed cadavers for surgery training. Anat. Histol. Embryol. 49, 372–381. doi: 10.1111/ahe.12539, PMID: 32059261

[ref98] Ventura-Antunes L. Mota B. Herculano-Houzel S. (2013). Different scaling of white matter volume, cortical connectivity, and gyrification across rodent and primate brains. Front. Neuroanat. 7:3. doi: 10.3389/fnana.2013.00003, PMID: 23576961 PMC3620553

[ref99] Viktorov I. V. Proshin S. S. (2003). Use of isopropyl alcohol in histological assays: dehydration of tissue, enbessing into paraffin, and processing of paraffin sections. Bull. Exp. Biol. Med. 136, 105–106. doi: 10.1023/a:1026017719668, PMID: 14534624

[ref100] Vonsattel J. P. Amaya Mdel P. Cortes E. P. Mancevska K. Keller C. E. (2008). Twenty-first century brain banking: practical prerequisites and lessons from the past: the experience of New York brain Bank, Taub institute, Columbia University. Cell Tissue Bank 9, 247–258. doi: 10.1007/s10561-008-9079-y, PMID: 18581261 PMC2847415

[ref101] Weisbecker V. (2011). Distortion in formalin-fixed brains: using geometric morphometrics to quantify the worst-case scenario in mice. Brain Struct. Funct. 217, 677–685. doi: 10.1007/s00429-011-0366-1, PMID: 22139139

[ref102] Wisniewski H. M. Wen G. Y. Kim K. S. (1989). Comparison of four staining methods on the detection of neuritic plaques. Acta Neuropathol. 78, 22–27. doi: 10.1007/bf00687398, PMID: 2472039

[ref103] Wolman M. (1958). Studies on the impregnation of nervous tissue elements. IV. Mechanism of impregnation of oligodendroglia and microglia. Lab. Investig. 7, 52–57. PMID: 13503199

[ref104] Xiao G. Li H. Zhao M. Zhou B. (2023). “Chapter eight - assessing metal ion transporting activity of ZIPs: intracellular zinc and iron detection” in Methods in enzymology. ed. Hu J., vol. 687 (Cambridge, MA: Academic Press), 157–184. 10.1016/bs.mie.2023.05.011 37666631

[ref105] Zachlod D. Kedo O. Amunts K. (2022). Anatomy of the temporal lobe: from macro to micro. Handb. Clin. Neurol. 187, 17–51. doi: 10.1016/b978-0-12-823493-8.00009-2, PMID: 35964970

