# Supplementary material for: Comparison of histological procedures and antigenicity of human post-mortem brains fixed with solutions used in gross anatomy laboratories
Source: Front Neuroanat. 2024 Apr 10;18:1372953. doi: 10.3389/fnana.2024.1372953 (PMC11039794; doi:10.3389/fnana.2024.1372953)

**Supplemental material**

Table 1 – Scores and data assessment for all variables of interest

| **Fixative** | **Sex** | **ID** | **EM** | **NU** | **CS** | **NeuN** | **GFAP** | **Iba1** | **PLP** | **CV** | | **LFB** | **PB** | **FB** | **BB** |
| --- | --- | --- | --- | --- | --- | --- | --- | --- | --- | --- | --- | --- | --- | --- | --- |
| NBF | F | 1 | 2 | 0 | 1 | 0 | 0 | 0 | 3 | | 2 | 0 | 2 | 2 | 1 |
|  |  | 2 | 1 | 0 | 1 | 1 | 2 | 2 | 3 | | 2 | 0 | 2 | 2 | 1 |
|  |  | 3 | 2 | 0 | 1 | 2 | 0 | 2 | 3 | | 2 | 0 | 2 | 2 | 2 |
|  |  | 4 | 3 | 1 | 1 | 2 | 2 | 2 | 2 | | 2 | 1 | 2 | 2 | 2 |
|  |  | 5 | 2 | 0 | 1 | 2 | 3 | 3 | 2 | | 2 | 0 | 2 | 2 | 1 |
|  | M | 6  7  8  9 | 3 | 1 | 1 | 3 | 1 | 3 | 2 | | 2 | 0 | 2 | 2 | 1 |
|  |  |  | 2 | 0 | 1 | 2 | 0 | 3 | 0 | | 2 | 0 | 2 | 2 | 1 |
|  |  |  | 3 | 1 | 1 | 2 | 1 | 2 | 2 | | 2 | 0 | 2 | 2 | 0 |
|  |  |  | 3 | 1 | 1 | 3 | 1 | 2 | 0 | | 2 | 1 | 2 | 1 | 2 |
|  |  | 10 | 2 | 1 | 1 | 0 | 2 | 2 | 3 | | 2 | 1 | 2 | 2 | 2 |
|  |  | 11 | 1 | 0 | 1 | 0 | 1 | 2 | 2 | | 2 | 0 | 2 | 1 | 1 |
|  |  | 12 | 1 | 1 | 1 | 3 | 2 | 2 | 1 | | 2 | 1 | 2 | 2 | 2 |
| SSS | F | 13 | 2 | 0 | 0 | 1 | 1 | 1 | 0 | | 2 | 2 | 2 | 2 | 1 |
|  |  | 14 | 1 | 0 | 0 | 2 | 3 | 3 | 3 | | 0 | 1 | 0 | 2 | 1 |
|  |  | 15 | NA | 0 | 0 | 2 | 3 | 3 | 3 | | 1 | 3 | 2 | 2 | 0 |
|  |  | 16 | 2 | 0 | 0 | 1 | 1 | 3 | 3 | | 1 | 1 | 1 | 1 | 2 |
|  | M | 17 | 3 | 0 | 0 | 1 | 2 | 2 | 0 | | 1 | 2 | 1 | 2 | 1 |
|  |  | 18 | 1 | 0 | 0 | 2 | 3 | 3 | 3 | | 1 | 2 | 2 | 1 | 1 |
|  |  | 19 | 1 | 1 | 0 | 2 | 3 | 3 | 3 | | 2 | 0 | 2 | 2 | 2 |
|  |  | 20 | 1 | 0 | 0 | 1 | 2 | 3 | 3 | | 1 | 2 | 2 | 1 | 1 |
|  |  | 21 | 2 | 1 | 0 | 1 | 3 | 2 | 2 | | 2 | 0 | 1 | 1 | 0 |
|  |  | 22 | 3 | 0 | 0 | 1 | 1 | 2 | 2 | | 2 | 1 | 1 | 1 | 1 |
|  |  | 23 | 1 | 1 | 0 | 2 | 1 | 2 | 3 | | 1 | 0 | 2 | NA | NA |
|  |  | 24 | 0 | 1 | 0 | 1 | 1 | 2 | 1 | | 2 | 1 | 2 | 0 | NA |
|  |  | 25 | 1 | 0 | 0 | 2 | 2 | 1 | 0 | | 2 | 2 | 2 | 1 | 0 |
| AFS | F | 26 | 0 | 0 | 1 | 3 | 3 | 3 | 3 | | 2 | 0 | 1 | 1 | 2 |
|  |  | 27 | 2 | 0 | 1 | 2 | 2 | 2 | 2 | | 0 | 3 | 1 | 2 | 1 |
|  | M | 28 | 1 | 1 | 1 | 3 | 3 | 3 | 3 | | 2 | 1 | 1 | 1 | 1 |
|  |  | 29 | 2 | 0 | 0 | 3 | 3 | 3 | 2 | | 2 | 0 | 2 | 1 | 0 |
|  |  | 30 | 1 | 0 | 0 | 2 | 1 | 3 | 3 | | 2 | 0 | 2 | 0 | NA |
|  |  | 31 | 3 | 0 | 1 | 1 | 3 | 3 | 3 | | 2 | 1 | 2 | 0 | NA |
|  |  | 32 | 2 | 1 | 1 | 3 | 2 | 3 | 2 | | 2 | 0 | 2 | 2 | 0 |
|  |  | 33 | 2 | 0 | 1 | 2 | 2 | 3 | 2 | | 2 | 3 | 2 | 1 | 2 |
|  |  | 34 | 2 | 1 | 1 | 3 | 3 | 3 | 2 | | 2 | 3 | 2 | 2 | 0 |
|  |  | 35 | 2 | 0 | 0 | 1 | 2 | 3 | 1 | | 1 | 2 | 2 | 1 | 2 |
|  |  | 36 | 1 | 1 | 0 | 3 | 3 | 3 | 3 | | 1 | 3 | 2 | 2 | 0 |
|  |  | 37 | 3 | 0 | 0 | 2 | 2 | 3 | 3 | | 1 | 2 | 1 | 1 | 1 |
|  |  | 38 | 2 | 0 | 1 | 3 | 2 | 3 | 2 | | 1 | 2 | 1 | 2 | 0 |
|  |  | 39 | 3 | 0 | 0 | 3 | 1 | 3 | 2 | | 2 | 1 | 2 | 1 | 0 |
|  |  | 40 | 2 | 0 | 0 | 3 | 2 | 1 | 2 | | 0 | 0 | 0 | 1 | 1 |
|  |  | 41 | 3 | 0 | 0 | 3 | 3 | 3 | 2 | | 2 | 1 | 2 | 1 | 0 |
|  |  | 42 | 3 | 1 | 1 | 3 | 3 | 3 | 2 | | 1 | 2 | 2 | 2 | 1 |

NA=Non-available, F=Female, M=Male, NBF=Neutral-Buffered formalin, SSS=Saturated-salt solution, AFS=Alcohol-formaldehyde solution

EM (Ease of manipulation): Categorical, 0=Very poor, 1=Poor, 2=Good, 3=Very good

NU (Neuropil Uniformity): Categorical, 0=Fissured, 1=Uniform

CS (Cellular Shape): Categorical, 0=Irregular, 1=Regular

NeuN, GFAP, Iba1, PLP: Antigenicity distribution for the respective antigen, 0=Absence, 1=Isolated cells, 2=Cell patches, 3=Homogeneous distribution

CV (Cresyl Violet): Categorical, 0=No staining, 1=Pale neurons, 2=Dark neurons

LFB (Luxol Fast Blue): Categorical, 0=No fibers and heterogenous blue stain, 1=Heterogenous fibers and differentiation, 2=Homogenous fibers but bad differentiation, 3=Homogenous fibers and good differentiation

PB (Prussian blue): Categorical, 0=No staining, 1=Pale neurons, 2=Dark neurons

FB (Fibers preservation of Bielchowsky’s staining): Categorical, 0=Absence, 1=Heterogeneous, 2=Homogeneous

BB (Bielchowsky’s background): Categorical, 0=Dark, 1=Intermediate, 2=Light

Figure 1 – Scatter dot plots of the variables of interest in relation to the PMI as a continuous variable


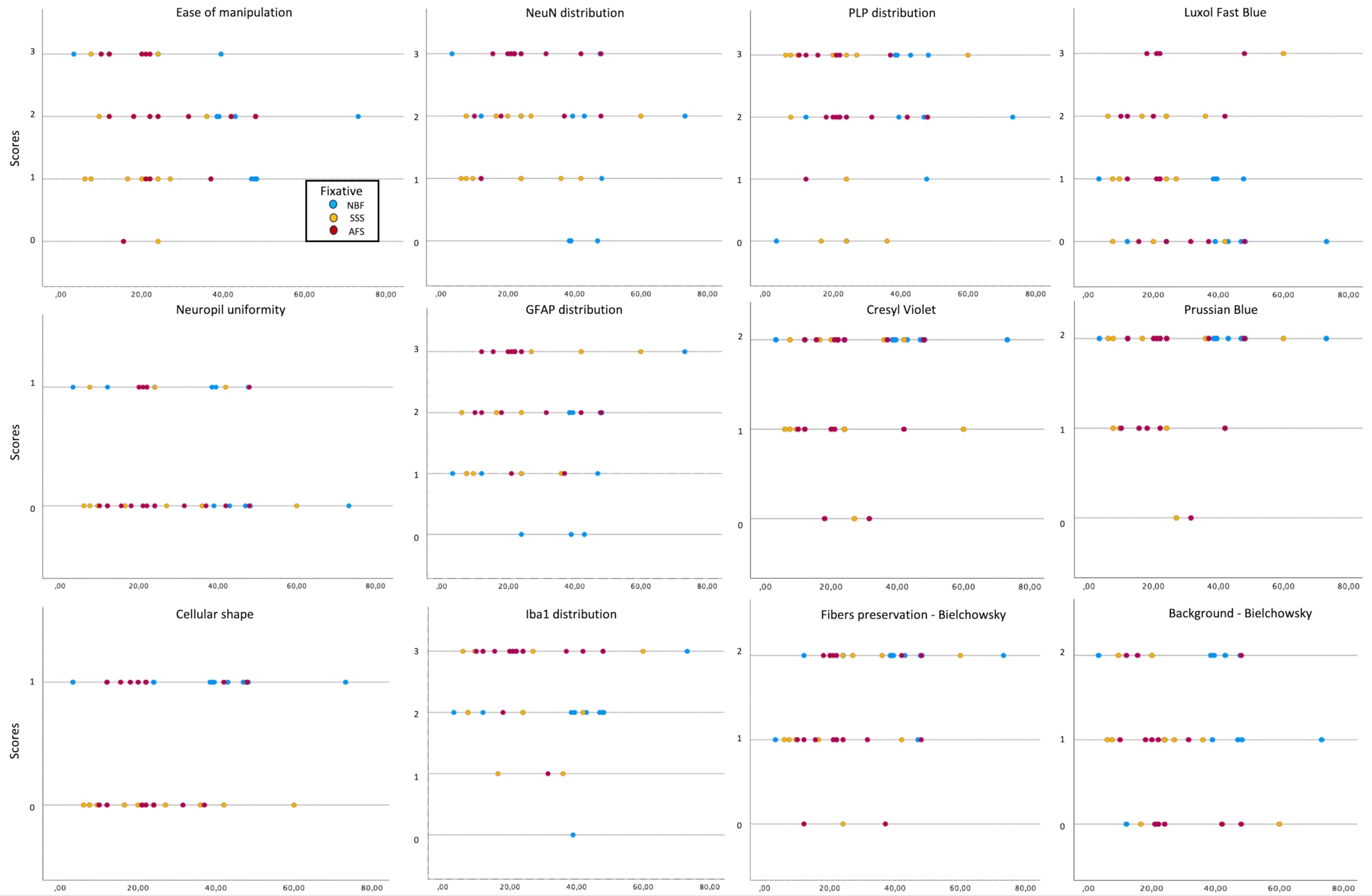


Figure 2 – Scatter dot plots of the variables of interest in relation to the HD as a continuous variable


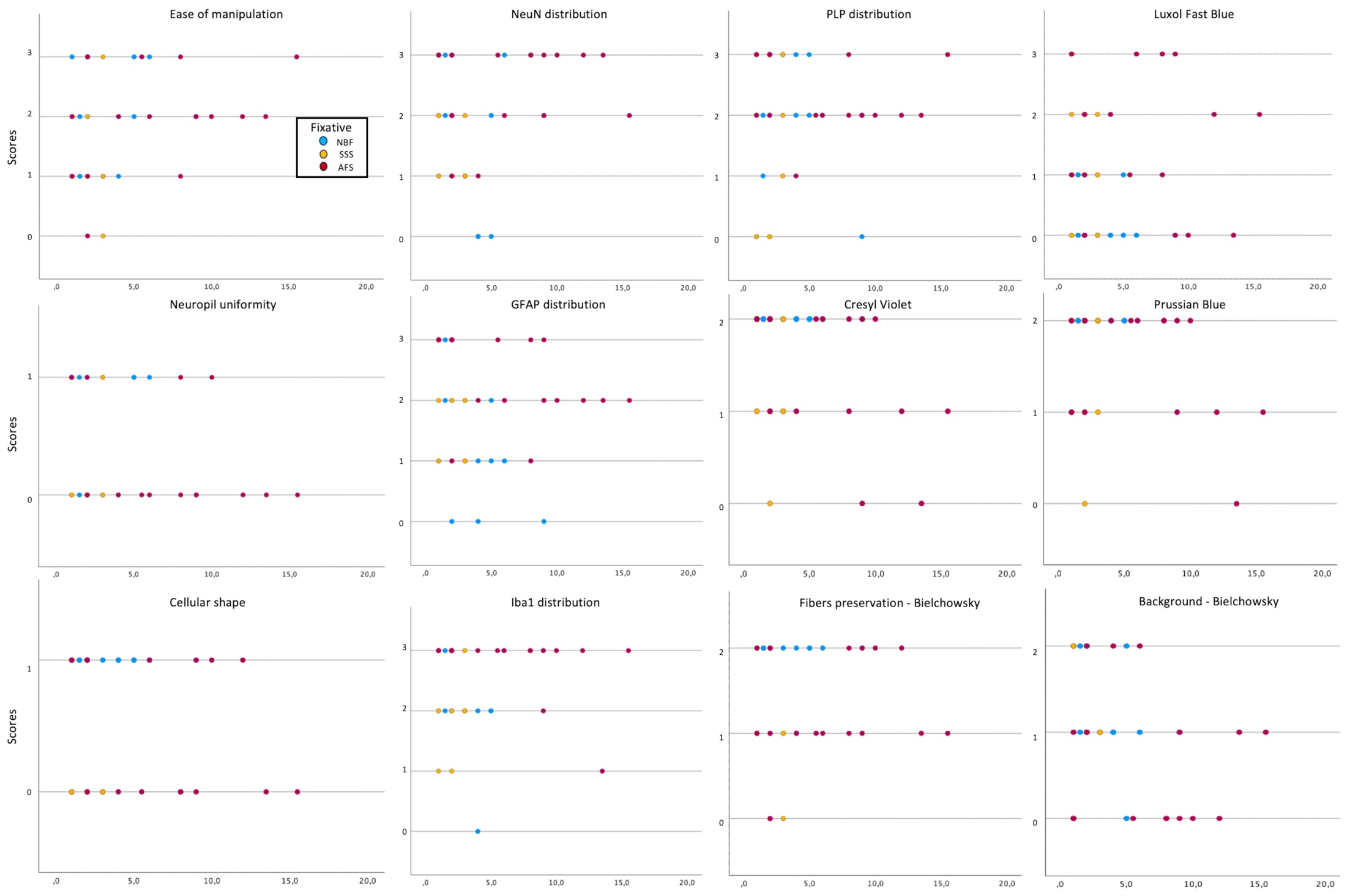

Supplement: Supplementary file 1 [file Data_Sheet_1.docx]
